# Unsymmetric Cisplatin-Based Pt(IV) Conjugates Containing a PARP-1 Inhibitor Pharmacophore Tested on Malignant Pleural Mesothelioma Cell Lines

**DOI:** 10.3390/molecules26164740

**Published:** 2021-08-05

**Authors:** Elisabetta Gabano, Giulia Pinton, Cecilia Balzano, Sara Boumya, Domenico Osella, Laura Moro, Mauro Ravera

**Affiliations:** 1 Dipartimento di Scienze e Innovazione Tecnologica, Università del Piemonte Orientale, Viale Michel 11, 15121 Alessandria, Italy; elisabetta.gabano@uniupo.it (E.G.); cecilia.balzano@uniupo.it (C.B.); domenico.osella@uniupo.it (D.O.); 2 Dipartimento di Scienze del Farmaco, Università del Piemonte Orientale, Largo Donegani 2/3, 28100 Novara, Italy; giulia.pinton@uniupo.it (G.P.); 20006489@studenti.uniupo.it (S.B.)

**Keywords:** cisplatin, Pt(IV) complexes, prodrugs, PARP-1 inhibitors, malignant pleural mesothelioma

## Abstract

Cisplatin is widely employed as a first-line chemotherapeutic agent for many solid tumors, including malignant pleural mesothelioma (MPM). However, its clinical use is limited by heavy side effects and acquired resistance, the latter being mainly related to enhanced DNA repair. Many clinical trials using combinations of platinum drugs and PARP-1 inhibitors (PARPis) have been carried out, with the hope that such combinations might lead to improved therapeutic efficacy against tumors. Here, the synthesis and efficacy in reducing MPM cell viability of four cisplatin-based Pt(IV) prodrugs containing the PARPi 3-aminobenzamide (3-ABA) fragment are described. The most promising conjugate is more effective than cisplatin or cisplatin/3-ABA combination, administered in equimolar doses, in inhibiting PARP-1 activity and inducing apoptosis in BRCA1/2 wild type MPM cells, grown as monolayer or as multicellular spheroids.

## 1. Introduction

Malignant pleural mesothelioma (MPM) is a rare and aggressive cancer the incidence of which dramatically increases in asbestos-polluted areas, as occurred in the town of Casale Monferrato (Northern Italy) due to the activity of the Eternit factory in the past [1]. The combination of the antifolate pemetrexed and a cytotoxic alkylating Pt(II)-based agent (cisplatin or carboplatin) has been established as the current standard of care, but only 40% of treated patients show a modest overall survival benefit [2]. In the absence of effective chemotherapeutic agents improving the efficacy of cisplatin, the management of MPM remains a challenge [3].

Cisplatin ((*SP-*4-2)-diamminedichloridoplatinum(II) (CDDP)) is the most widely used agent in combination chemotherapy of solid tumors [4,5]. Its antitumor activity mainly relies on the ability to make adducts with DNA, blocking replication and transcription, halting tumor growth, and leading to cell apoptosis. Regrettably, its mechanism of action is highly nonspecific, and its clinical application has limitations due to heavy side effects and induction of chemoresistance [6]. Enhanced DNA repair is one of the mechanisms responsible for acquired cisplatin resistance [7].

Poly(ADP-ribose) polymerases (PARPs) are a group of nuclear enzymes that play an important role by catalyzing the poly(ADP-ribosyl)ation of a number of acceptor proteins (PARylation). PARylation is the crucial post-translational modification that occurs during the response to multiple types of DNA damage. PARP-1, the first member identified of this family, is well-known for its role in the base excision repair pathway for DNA single-strand breaks (SSBs) following DNA damage by exogenous genotoxic substances [8]. Failure to repair SSBs leads to replication fork collapse and formation of double-strand breaks (DSBs). DSBs and PARP trapping are lethal in homologous recombination (HR)-deficient cancer cells. Therefore, PARP-1 inhibitors (PARPis), either as a single drug or in combination with other chemotherapeutic agents, are being extensively explored in relation to tumors bearing defects in HR pathways, such as breast and ovarian with BRCA1/2 mutations. Even some MPM patients (about 10%) display pathogenic truncating variants (PTVs) in DNA repair genes (including BRCA1 and BRCA2), whereas no healthy controls show similar mutations [9]. The first results from a clinical trial performed to test the sensitivity to PARP-1 inhibition of BAP1- or BRCA1-deficient mesotheliomas have been published very recently [10].

Furthermore, it has also become apparent that the strategy to combine a PARPi with a DNA-damaging agent can expand the use of PARPis to a greater range of cancer types, independently of their BRCA status. The therapeutic efficacy of combining PARPis with DNA-damaging drugs, including metal drugs, has proven to be effective in some preclinical and early trial data [11,12,13,14,15,16,17]. In addition, PARP-1 inhibition ameliorated chemotherapy-induced neuropathy and gastrointestinal dysfunction. Therefore, co-treatment of cisplatin with PARPis could also be a powerful tool in alleviating its side effects [18].

**Scheme 1:**
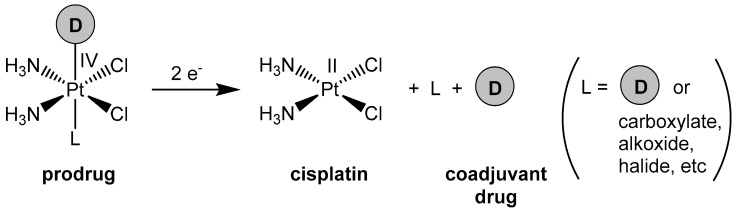
Reduction reaction of a dual-action cisplatin-based Pt(IV) prodrug (activation by reduction).

Another family of Pt-based anticancer drug candidates is represented by the octahedral Pt(IV) derivatives (e.g., satraplatin). These complexes, obtained from square planar Pt(II)-based drugs by addition of two axial ligands via chemical oxidation and then conjugation, have been actively studied. They act as prodrugs since they are (ideally) only reduced in the hypoxic tumor microenvironment and release the cytotoxic cisplatin metabolite plus the two axial ligands (Scheme 1). These axial ligands can consist of one or two molecules of a biologically inactive moiety (L) or, more interestingly, of another drug (**D**). As regards the case of unsymmetric Pt(IV) prodrugs, ^§^ (^§^ We prefer to use the term “symmetric” when the two axial ligands are identical and “unsymmetric” when they are different, rather than “asymmetric” or “non-symmetric”, which imply that all symmetry elements have been lost) provided that **D** is a synergistic (or adjuvant) agent with respect to cisplatin, the final bifunctional chemotherapeutic agent should produce a dual-potency effect (note: we prefer to use the term “symmetric” when the two axial ligands are identical and “unsymmetric” when they are different, rather than “asymmetric” or “non-symmetric”, which imply that all symmetry elements have been lost) [19,20,21,22,23,24]. The “innocent” ligand L****, which occupies the second axial position of Pt(IV), must be carefully chosen to confer the optimal balance between lipophilicity, water solubility, and stability with respect to premature reduction and hydrolysis of the conjugate. Moreover, the enhanced lipophilicity of the final Pt(IV) conjugate with respect to its components causes higher cell uptake (synergistic cellular accumulation), thus favoring the overall activity [23]. Another typical feature of such conjugates is the release of the two drugs at the same time and in a precise (stoichiometric) ratio. This can represent an advantage or disadvantage, depending on the optimal scheduling identified for the drug combination under study.

The treatment with cisplatin-based Pt(IV) conjugates bearing a PARPi in the axial position (as **D**) is worthy of investigation. The released cisplatin causes its cytotoxic effect via the formation of DNA adducts, and a PARPi should block or at least minimize DNA repair. This should result in an accumulation of DNA damages and induction of strong pro-apoptotic signals [25].

**Figure 1:**
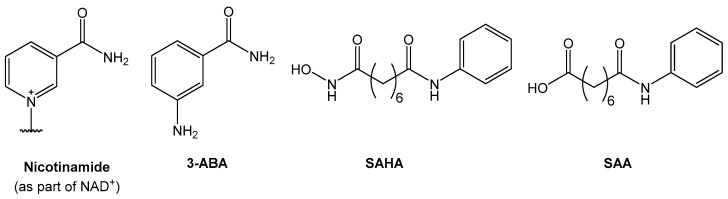
Sketch of the nicotinamide part of NAD^+^ and some molecules cited in the text (3-aminobenzamide, **3-ABA**; suberoylanilide hydroxamic acid or vorinostat, **SAHA**; 8-oxo-8-(phenylamino)octanoato, **SAA**).

Since PARP uses nicotinamide adenine dinucleotide (NAD^+^) as a substrate, a PARPi should compete with NAD^+^ at the level of the catalytic domain. Among the PARPis so far developed, various benzamide derivatives, structurally similar to nicotinamide, resulted in activity in the low μM range. The substitution in position 3 of the aryl ring with electron-donating groups generally increases the potency; accordingly, 3-aminobenzamide (**3-ABA**, Figure 1) showed good inhibitory activity [26,27]. Importantly, **3-ABA** amplifies the cellular sensitivity to cisplatin in vitro [28,29,30,31].

**Figure 2:**
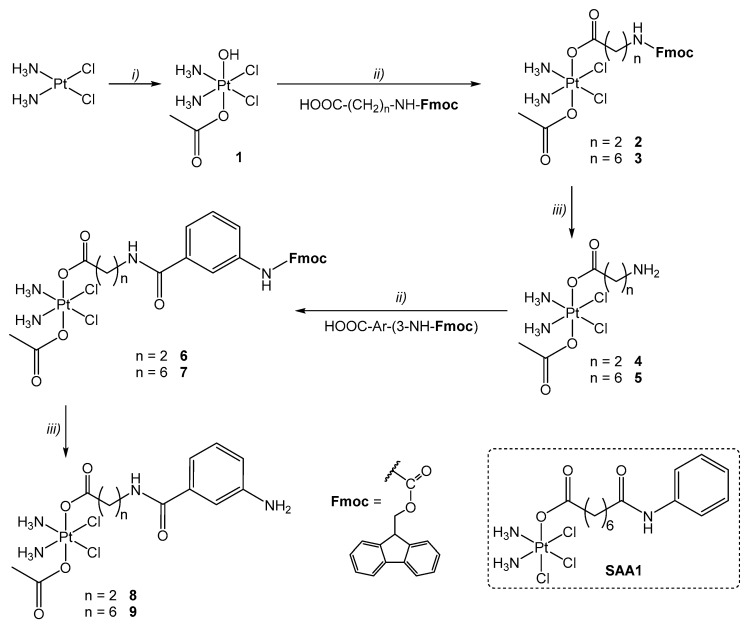
Scheme of the reaction pathways for the synthesis of the compound under investigation. *(i)* H_2_O_2_, acetic acid; *(ii)* HATU, DIPEA, DMF; *(iii)* piperidine, DMF. DIPEA = *N*,*N*-diisopropylethylamine; DMF = *N*,*N*-dimethylformamide; HATU = 1-[bis(dimethylamino)methylene]-1*H*-1,2,3-triazolo[4,5-b]pyridinium 3-oxide hexafluorophosphate.

Recently, Gou et al. reported on a cisplatin-based Pt(IV) prodrug, namely **SAA1** (Figure 2), containing the preformed 8-oxo-8-(phenylamino)octanoato (**SAA**, Figure 1) ligand in axial position. **SAA** is structurally similar to suberoylanilide hydroxamic acid or vorinostat (**SAHA**), a potent histone deacetylases (HDAC) inhibitor [32]. Even though the Ar-N(H)-C(O)- portion of **SAA** is a congener of acetanilide and not of benzamide (Ar-C(O)-N(H)-) and the aryl ring is deprived of any substituent in 3-position, the **SAA** ligand and its Pt(IV) conjugate **SAA1** inhibited PARP-1 activity in HeLa cells. Interestingly, CDDP resulted in a deprivation of any PARP-1 inhibition propensity; thus, the **SAA** ligand only, released upon reduction, brought about such an inhibition. **SAA1** exhibited antiproliferative activity at a slightly lower level than that of CDDP against a panel of human tumor cells, but its cytotoxicity had excellent results on the gastric cancer cell line SGC790 and on its cisplatin-resistant subline, thus overcoming chemoresistance [32].

More recently, Zhu et al. have synthesized and tested seven unsymmetric cisplatin-based Pt(IV) conjugates containing preformed (potential) PARPis. Most of their structures were based on phthalazinone and inspired by the FDA-approved PARPis olaparib and talazoparib. The synthesized ligands and conjugates showed a PARP inhibitory effect; moreover, comparable or improved activity vs. CDDP on a panel of human cancer cell lines was observed [33].

A novel approach for the synthesis of Pt(IV)–carbamato complexes has been applied to obtain some Pt(IV)-**3-ABA** conjugates. The cytotoxicity data did not show synergistic effects but, unfortunately, PARPi activity was not evaluated [34].

This lays the basis for the synthesis of the Pt(IV) conjugates **6**–**9** containing the PARPi **3-ABA** leitmotif (Figure 2) to be tested on a panel of highly chemo-insensitive MPM cells.

## 2. Results and Discussion

### 2.1. Synthesis of the Bifunctional Pt(IV) Prodrugs

The Pt(IV)-**3-ABA** carbamate, albeit very elegant from a chemical point of view, did not result in a very effective compound. For this reason, a slightly more complex synthetic strategy was attempted. Complexes **6**–**9** were designed to generate the **3-ABA** functionality at the end of an organic chain already coordinated as an axial ligand (Figure 2). The complexes differ in the length of the [CH_2_]_n_ arm (*n* = 2 or 6) which links the carboxylate functionality anchored to the Pt(IV) core and the terminal **3-ABA** moiety. An “innocent” (biologically inactive) acetato ligand was used for the second axial position of Pt(IV), with the aim of conferring higher stability with respect to reduction and hydrolysis than the chlorido ligand present in **SAA1** [35,36].

The starting point for the synthesis of all the complexes is the synthon (*OC*-6-44)-acetato-diammine-dichlorido-hydroxido-platinum (IV) (**1**), obtained by oxidizing CDDP with H_2_O_2_ in acetic acid [37]. The fluorenyl-methyl-oxy-carbonyl (Fmoc)-protected 3-aminopropanoic acid [38,39] and 7-aminoheptanoic acid were activated by using 1-[bis(dimethylamino)methylene]-1*H*-1,2,3-triazolo[4,5-b]pyridinium 3-oxide hexafluorophosphate (HATU) in the presence of *N,N*-diisopropylethylamine (DIPEA). The resulting activated acids underwent the substitution reaction with the hydroxide ligand on **1** to afford **2** or **3**. Then, the two complexes were Fmoc-deprotected with piperidine [38], giving the intermediates **4** [38,39] and **5**. By using the same coupling agents (i.e., HATU and DIPEA), **4** and **5** were reacted with 3-(Fmoc-amino)benzoic acid, affording **6** and **7**. Finally, the Fmoc-protected complexes **6** and **7** were deprotected with piperidine, giving **8** and **9** (see the Material and Methods section and the Supplementary Materials for details). In spite of the several protection/deprotection procedures, the yields of **6**–**9** were acceptable (45–59% from **1**).

To verify the activation by reduction mechanism, complexes **6**–**9** were challenged with the model bioreductant ascorbic acid (AA, 10 mM in water) in HEPES buffer (HEPES = 4-(2-hydroxyethyl)-1-piperazineethanesulfonic acid, 50 mM, pH = 7.4) at 37 °C. The reaction was monitored by following the RP-HPLC peaks over 24 h. The different solubility and retention times required different experimental conditions for the different compounds (cosolvent DMF or MeOH was necessary to maintain the Pt compounds in solution, >5% *v*/*v*). For these reasons, complete reduction for **8** and **9** was observed after 8 h, whereas for **6** and **7**, the >85% peak area disappeared after 24 h only. Despite the different reaction times, this experiment demonstrates that AA is able to reduce **6**–**9**. Moreover, it is important to recall that derivatives of the parent complex **4** have been more quickly reduced to CDDP in the presence of cell cytosol extract [38,39]. Complexes **6**–**9**, having very similar coordination skeletons, can be expected to behave in the same way. The bulk of these data should confirm the hypothesized mechanism.

### 2.2. Evaluation of the Antiproliferative Activity

Complexes **6**–**9** were tested on a panel of human MPM cell lines together with CDDP and **SAA1**, added for comparison (Table 1). The half-maximal inhibitory concentration, IC_50_, was determined on MSTO-211H (biphasic phenotype), REN (epithelioid), BR95 (epithelioid), and MM98 (sarcomatoid) cancer cell lines to identify the most promising Pt(IV) conjugate to be further investigated. To determine the number of viable cells, the use of the dye exclusion assay was preferred to the colorimetric assay because of the heterogeneity in the energy metabolism between the different MPM cell lines. This may justify minor differences with respect to the previously published IC_50_ for CDDP [40].

**3-ABA** is known to have negligible antiproliferative activity alone; in fact, it showed IC_50_ = 5412 ± 28 μM on MSTO-211H. Surprisingly, the bulky Fmoc-protected complexes **6** and **7** appeared to be more active than their deprotected counterparts **8** and **9**. Furthermore, the increase of the -(CH_2_)_n_- spacer from *n* = 2 to 6 was detrimental for the antiproliferative activity, **7** being less active than **6** and **9** less active than **8**.

**SAA1** showed slightly lower potency than CDDP on all MPM cell lines, as in the previous investigation by Gou et al. on different human tumor cell lines [31].

The ratio between the IC_50_ of CDDP and the IC_50_ of the Pt(IV) complexes indicates that **6** is slightly more cytotoxic than CDDP on all cell lines (ratio around 2), just overcome by **7** on sarcomatoid MM98 only.

### 2.3. Cell Uptake

Since it has been reported that Pt(IV) complexes enter cells by passive diffusion only [41], with a few exceptions [42], the evaluation of lipophilicity is key because it is related to the ability of a molecule to passively cross cellular membranes. RP-HPLC techniques are often used to measure the lipophilicity of a compound, since the retention is due to partitioning between the C18 chains of the stationary phase (representing the cellular membrane) and the aqueous eluent (representing the water inside and outside cells) [43,44]. For this purpose, the retention times (*t*_R_) of the complexes were transformed as log *k*′, where *k*′ = (*t*_R_ − *t*_0_)/*t*_0_) and *t*_0_ is the column dead-time (Table 1). The data follow the same trend as the lipophilicity parameters calculated for the free ligands alone (VCCLAB ALOGPS 2.1 log *P*_o/w_ = 3.73, 4.96, 0.04, and 2.21 for the non-acetato axial ligand of **6**, **7**, **8**, and **9**, respectively, in the form of methyl esters) [45].

Cellular accumulation of compounds **6**–**9** was evaluated by means of the intracellular Pt uptake after 4 h treatment of MSTO-211H cells and expressed as ng Pt per 10^6^ cells (Table 1). As expected, the Fmoc protection of **6** and **7** enhances the lipophilicity of the complexes, facilitating their cell uptake. Also the elongation of the organic linker from -(CH_2_)_2_- to -(CH_2_)_6_- increases (to a minor extent) the lipophilicity in **7** and **9** with respect to **6** and **8**.

The behavior of CDDP is apparently incongruous: its log *k′* value, which is similar to that of **8** and **9**, does not fit with its higher uptake, nearer to **6** and **7**. However, this is in tune with studies from the literature suggesting that, together with passive diffusion, active mechanisms (i.e., via the copper transporter CTR1) further contribute to the cell uptake of CDDP [46,47].

### 2.4. Verification of the Targets of ***6***

**Figure 3:**
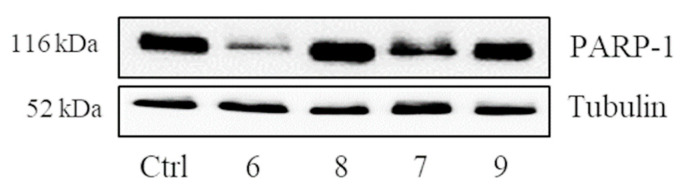
Western blot analysis of PARP-1 expression in MSTO-211H cells (whole cell lysate) after treatment (72 h) with diluent (Ctrl) or compounds **6**–**9** at their IC_50_ values. Tubulin was used as loading control.

It is necessary to verify how much the presence of the bulky Fmoc group might affect the recognition of the PARP catalytic domain. It is important to recall that all PARPis designed as analogues of nicotinamide must contain a primary or secondary amide. Indeed, this is the case of **6** [27]. The effects of compounds **6**–**9** on the expression of PARP-1 were evaluated by treating MSTO-211H cells for 72 h with equitoxic (at their IC_50_) concentrations of complexes, followed by Western blot analysis of whole cell lysate. Figure 3 shows that treatment with the Fmoc-protected **6** resulted in lower expression of PARP-1 in the series, followed by the Fmoc-protected **7**, whereas Fmoc-deprotected **8** and **9** were similar to the untreated control. First of all, the higher cellular uptake of **6** and **7** increases the intracellular concentration of the Pt metabolite and, in particular, of the ligands, potentially PARPis (synergistic cellular accumulation) [48]. Additionally, this result demonstrates that **6**, and to a lesser extent **7**, downregulate PARP-1 expression. The PARP-1 pharmacophore includes, among the other features, an amide moiety, and the presence of large groups in the structure of the molecule may improve potency [27]. Although we considered the synergistic accumulation at the basis of the improved PARPi properties of the Fmoc-containing compounds, we cannot rule out the possibility that the fluorenylmethoxycarbonyl-protecting group could play a role.

**Figure 4:**
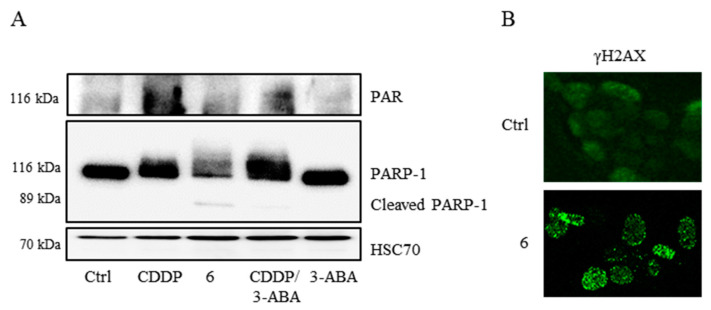
(**A**) Representative Western blot showing poly(ADP-ribose) (PAR) and PARP-1 expression following 72 h MSTO-211H cell treatment with diluent (Ctrl) or 5 µM concentrations of cisplatin (CDDP), **6**, CDDP + **3-ABA** combination (1:1 ratio), and **3-ABA** alone. HSC70 protein was used as loading control. (**B**) Immunocytochemistry was used to capture representative γH2AX focus images following 72 h MSTO-211H cell treatment with diluent (Ctrl) or 5 µM of complex **6**.

The effect of the most promising complex **6** on PARP-1 activity was further evaluated. MSTO-211H cells were treated with equimolar concentrations (5 µM) of **6**, CDDP, and **3-ABA** for 72 h. The two last compounds were used either alone or in combination in a 1:1 molar ratio. Western blot analysis evaluated the expression of poly(ADP-ribose) (PAR), which is the product of PARP-1 activity (Figure 4A). An increase in PARP-1 parylation was observed in response to CDDP treatment and to a lesser extent in response to treatment with the mixture of CDDP and **3-ABA**. No self-parylation of PARP-1 was evidenced in cells treated with **6** or **3-ABA**. At this dose and timing CDDP did not induce PARP-1 cleavage but only its activation, whereas complex **6** alone did, suggesting induction of apoptosis in MSTO-211H cells.

DNA damage, evidenced as an increased number of γH2AX foci in cells treated with complex **6**, confirmed that the platinum moiety contributed to the high cytotoxicity of the complex (Figure 4B; γH2AX is a sensitive molecular marker of DNA damage). This is in tune with the activation by reduction mechanism (Scheme 1) that is able to generate CDDP in the cells. As expected, the produced CDDP damaged DNA, as shown in Figure 4B.

**Figure 5:**
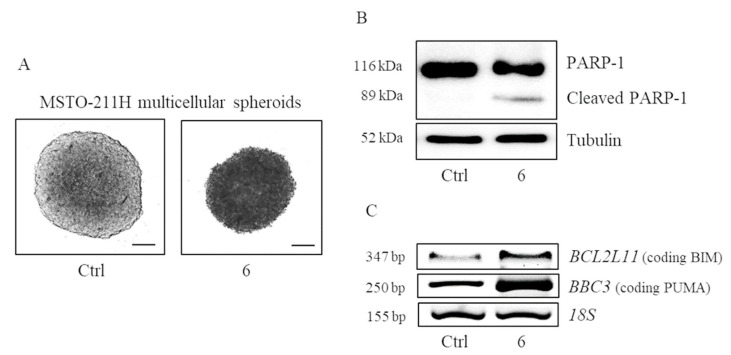
Compound **6** induces apoptosis in MSTO-211H multicellular spheroids (MCS). (**A**) Representative microscope images of MCS treated for 72 h with diluent (Ctrl) or 10 μM **6**. Scale bar = 100 μm. (**B**) Representative Western blot showing PARP-1 cleavage in MSTO-211H MCS treated with **6**. Tubulin was used as loading control. (**C**) RT-PCR showing induction of BCL2L11 and BBC3 in MSTO-211H MCS treated with **6**. 18S was used as housekeeping gene.

The capability of compound **6** to induce apoptosis was confirmed also when MSTO-211H cells were cultured as multicellular spheroids (MCSs). MCSs and other similar 3D cultures represent an improvement of classical 2D conditions, having a more complex architectural structure and better bio-mimicking of the in vivo microenvironment. Although they suffer many of the limitations of the 2D cultures, they are a powerful tool to narrow down the gap between the in vitro and in vivo models. Interestingly, it has been demonstrated that spheroid cultures recapitulate the resistance of mesothelioma cells to apoptosis more effectively than 2D cultures [49,50]. In MCS treated with **6**, apoptotic death was evidenced by altered spheroid morphology (Figure 5A), PARP-1 cleavage (Figure 5B), and induction of the genes encoding pro-apoptotic activators BIM and PUMA (Figure 5C).

In order to confirm that PARP-1 is the direct target of **6**, the cellular thermal shift assay (CETSA^®^) was employed. This assay is based on the biophysical principle of ligand-induced thermal stabilization of target proteins. Indeed, ligand-bound proteins are stabilized by their interacting partner and melt at a higher temperature with respect to the native protein. The test involves the drug treatment of the cellular system of choice, the heating of the cell to different temperatures, and the quantification of the presence of target protein in the soluble fraction by Western blotting to validate and quantify target engagement of drugs in cells and tissues [51].

**Figure 6:**
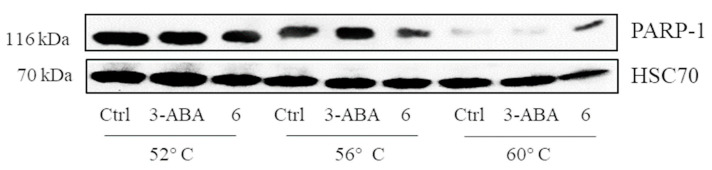
Cellular thermal shift assay (CETSA^®^) for in-cell PARP-1 target engagement. Representative Western blot showing thermostable PARP-1 following indicated heat shocks (range of 52–60 °C) in MSTO-211H cells, untreated (Ctrl) or treated with **6** or **3-ABA** (50 µM) for 15 min. HSC70 was used as loading control.

Multiple aliquots of MSTO-211H intact cells, previously incubated with the diluent, **3-ABA** or **6**, were heated to different temperatures ranging from 48 °C to 60 °C. After lysis, the soluble fractions were analyzed by SDS-PAGE followed by Western blot. In Figure 6, the stabilities of **6** and **3-ABA**, at 52, 56, and 60 °C, are compared. PARP-1 protein levels from cells incubated with **6** were more stable to heating as compared with the control and **3-ABA** groups, indicating potent binding of **6** to PARP-1.

## 3. Materials and Methods

### 3.1. Chemical Procedures

All the chemicals (Alfa Aesar-Thermo Fisher GmbH, Kandel, Germany and Sigma Aldrich-Merck KGaA, Darmstadt, Germany) were used without further purification. Complexes (*OC*-6-44)-acetato-diammine-dichlorido-hydroxido-platinum (IV), **1 [37]**, (*OC*-6-44)-acetato-(β-alaninato)-diammine-dichlorido-platinum (IV), **4**, and its Fmoc-protected form **2 [38]**, were prepared according to already published procedures. **SAA1** was a generous gift of Professor Shaohua Gou (Southeast University, Nanjing, China) [32].

The purity of all the compounds was routinely verified by analytical RP-HPLC (see below) and elemental analysis. Elemental analyses were carried out with an EA3000 CHN Elemental Analyzer (EuroVector, Milano, Italy).

^1^H, ^13^C, and ^195^Pt mono- and bi-dimensional NMR spectra were recorded with a Bruker Avance III 500 MHz instrument at 500 (^1^H), 125.7 (^13^C), and 107.2 (^195^Pt) MHz, respectively. For the NMR numbering schemes, see the Supplementary Materials. The solvent residual peak was used as internal reference for ^1^H and ^13^C, whereas ^195^Pt chemical shifts were referenced to an external solution of K_2_[PtCl_4_] in saturated aqueous KCl. The shift for K_2_[PtCl_4_] was adjusted to −1628 ppm from Na_2_PtCl_6_.

RP-HPLC analyses were performed on a Waters HPLC-MS instrument (equipped with Alliance 2695 separations module, 2487 dual lambda absorbance detector set at 210 nm, and 3100 mass detector). Mass spectra were recorded using source and desolvation temperatures set to 150 and 250 °C, respectively, with N_2_ used both as a drying and as a nebulizing gas. The cone and the capillary voltages were usually +30 V and 2.70 kV, respectively. Quasi-molecular ion peaks [M + H]^+^ were assigned on the basis of the *m*/*z* values and the simulated isotope distribution patterns.

### 3.2. Synthesis of the Pt(IV) Prodrugs

#### 3.2.1. Synthesis of (*OC*-6-44)-Acetato-[7-(fmoc-amino)-heptanoato]-diammine-dichlorido-platinum(IV), **3**

In a 10 mL round-bottom flask, 0.250 g of **1** (0.664 mmoL), 0.163 g of 7-(Fmoc-amino)heptanoic acid (0.444 mmoL), and 0.253 g of coupling agent (1-[bis(dimethylamino)methylene]-1*H*-1,2,3-triazolo[4,5-b]pyridinium 3-oxide hexafluorophosphate) (HATU, 0.665 mmoL) were suspended in about 3 mL of anhydrous *N*,*N*-dimethylformamide (DMF). Then, 116 µL of *N*,*N*-diisopropylethylamine (DIPEA, 0.662 mmoL) was added to the yellowish suspension, which turned orange. The reaction mixture was left to be stirred overnight at room temperature in the dark. The resulting suspension was centrifuged to remove unreacted solid species. The supernatant solution was dried under reduced pressure, obtaining a yellow oil. This residue was recrystallized with dichloromethane and diethylether until the precipitation of a yellow solid. Then, the solid was introduced in a centrifuge tube and washed four times with diethylether, five times with 1% *w*/*w* formic acid to remove DIPEA, and twice with cold ultrapure water. The solid was dried under a gentle nitrogen flow, obtaining 0.209 g of brownish powder (yield 65%). Anal. calcd. for C_24_H_33_Cl_2_N_3_O_6_Pt: C, 39.73; H, 4.58; N, 5.79; found: C, 40.08; H, 4.26; N, 6.04. ESI-MS (positive ion mode): 726 *m*/*z*; calcd. for C_24_H_34_Cl_2_N_3_O_6_Pt [M + H]^+^ 726 *m*/*z*. ^1^H NMR (CD_3_)_2_SO) *δ*: 1.24 (m, 4H, H_AH_4 and H_AH_5), 1.37 (m, 2H, H_AH_6), 1.44 (m, 2H, H_AH_3), 1.90 (s, 3H, CH_3-Ac_), 2.20 (t, *J*^3^ = 6.9 Hz, 2H, H_AH_2), 2.96 (m, 2H, H_AH_7), 4.20 (t, *J*^3^ = 6.8 Hz, 1H, H_F_3), 4.29 (d, *J*^3^ = 6.8 Hz, 2H, H_F_2), 6.52 (m, 6H, NH_3_), 7.24 (t, *J*^3^ = 5.6 Hz, 1H, NH_βA_), 7.32 (t, *J*^3^ = 7.4 Hz, 2H, H_F_6), 7.35 (t, *J*^3^ = 7.4 Hz, 2H, H_F_7), 7.42 (d, *J*^3^ = 7.4 Hz, 2H, H_F_5), 7.88 (d, *J*^3^ = 7.4 Hz, 2H, H_F_8) ppm; ^13^C NMR ((CD_3_)_2_SO) *δ*: 22.9 (CH_3-Ac_), 25.4 (C_AH_3), 26.1 and 28.3 (C_AH_4 and C_AH_5), 29.3 (C_AH_6), 35.7 (C_AH_2), 39.5 (C_HA_1 under (CD_3_)_2_SO), 46.8 (C_F_3), 65.1 (C_F_2), 120.1 (C_F_8), 125.1 (C_F_5), 127.0 (C_F_6), 127.6 (C_F_7), 140.7 and 143.9 (C_F_4 and C_F_9), 156.2 (C_F_1), 178.3 (C(O)_Ac_), 180.8 (C_AH_1) ppm; ^195^Pt NMR ((CD_3_)_2_SO) *δ*: 1222 ppm.

#### 3.2.2. Synthesis of (*OC*-6-44)-Acetato-(7-aminoheptanoato)-diammine-dichlorido-platinum(IV), **5**

In a 10 mL round-bottom flask, 0.100 g of **3** (0.138 mmoL) was added to 3 mL of a 6.7% solution of piperidine in DMF (200 µL in 2.8 mL), obtaining a yellow solution. After 1 h of stirring at room temperature in the dark, the excess solvent was removed under reduced pressure, obtaining a brown solid that was initially washed with a few drops of acetone and 2 mL of diethylether and then with diethylether (four times), chloroform (four times), and once again with diethylether. Finally, the solid was dried under a gentle nitrogen flow, obtaining 0.068 g of brown powder (yield 98%). Anal. calcd. for C_9_H_23_Cl_2_N_3_O_4_Pt: C, 21.48; H, 4.61; N, 8.35; found: C, 21.67; H, 4.86; N, 8.07. ESI-MS (positive ion mode): 504 *m*/*z*; calcd. for C_9_H_24_Cl_2_N_3_O_4_Pt [M + H]^+^ 504 *m*/*z*. ^1^H NMR ((CD_3_)_2_SO) *δ*: 1.27 (m, 4H, H_AH_4 and H_AH_5), 1.47 (m, 4H, H_AH_3 and H_AH_6), 1.90 (s, 3H, CH_3-Ac_), 2.19 (t, *J*^3^ = 6.9 Hz, 2H, H_AH_2), 2.71 (m, 2H, H_AH_7), 6.56 (m, 6H, NH_3_) ppm; ^13^C NMR ((CD_3_)_2_SO) *δ*: 22.8 (CH_3-Ac_), 25.2, 25.7 and 28.0 (C_AH_3, C_AH_4, C_AH_5, and C_AH_6), 35.6 (C_AH_2), 39.5 (C_AH_7, under (CD_3_)_2_SO, 178.2 (C(O)_Ac_), 180.9 (C_AH_1) ppm; ^195^Pt NMR ((CD_3_)_2_SO) *δ*: 1225 ppm.

#### 3.2.3. Synthesis of (*OC*-6-44)-Acetato[3-(3-(fmoc-amino)-benzamido)-propanoato]-diammine-dichlorido-platinum(IV), **6**

In a 10 mL round-bottom flask, 0.100 g of **4** (0.224 mmoL), 0.096 g of 3-(Fmoc-amino)benzoic acid (0.252 mmoL), and 0.127 g of the coupling agent HATU (0.334 mmoL) were suspended in 3 mL of anhydrous DMF. Then, 50 µL of DIPEA (0.286 mmoL) was added to the suspension, which turned from yellow to orange. The reaction mixture was left to be stirred overnight at room temperature in the dark until an orange solution was obtained. The excess of solvent was removed under reduced pressure. The resulting orange oil was recrystallized with dichloromethane and diethylether until the precipitation of a yellow-brown solid. Then the solid was introduced in a centrifuge tube and washed four times with diethylether, five times with 1% *w*/*w* formic acid in order to remove DIPEA, and twice with cold ultrapure water. The solid was dried under a gentle nitrogen flow, obtaining 0.153 g of yellowish powder (yield 87%). Anal. calcd. for C_27_H_30_Cl_2_N_4_O_7_Pt: C, 41.13; H, 3.83; N, 7.11; found: C, 41.39; H, 4.05; N, 6.91. ESI-MS (positive ion mode): 789 *m*/*z*; calcd. for C_27_H_31_Cl_2_N_4_O_7_Pt [M + H]^+^ 789 *m*/*z*. ^1^H NMR ((CD_3_)_2_SO) *δ*: 1.91 (s, 3H, CH_3-Ac_), 2.47 (m, 2H, H_βA_2 or H_βA_3), 3.43 (m, 2H, H_βA_2 or H_βA_3), 4.31 (t, *J*^3^ = 6.6 Hz, 1H, H_F_3), 4.47 (d, *J*^3^ = 6.7 Hz, 2H, H_F_2), 6.53 (m, 6H, NH_3_), 7.35 (t, *J*^3^ = 7.4 Hz, 4H, H_F_6), 7.43 (t, *J*^3^ = 7.4 Hz, 4H, H_F_7), 7.76 (d, *J*^3^ = 7.4 Hz, 2H, H_F_5), 7.91 (d, *J*^3^ = 7.4 Hz, 2H, H_F_8), 7.35–7.91 (m, 4H, H_Bz_3, H_Bz_5, H_Bz_6, and H_Bz_7), 8.28 (t, *J*^3^ = 5.5 Hz, 1H, NH_βA_), 9.84 (s, 1H, NH_Bz_) ppm; ^13^C NMR ((CD_3_)_2_SO) *δ*: 22.9 (CH_3-Ac_), 35.9 and 36.3 (C_βA_2 and C_βA_3), 46.6 (C_F_3), 65.7 (C_F_2), 120.2 (C_F_8), 125.1 (C_F_5), 127.7 and 127.1 (C_F_6 and C_F_7), 117.6, 120.9–121.0 and 128.6 (C_Bz_3, C_Bz_5, C_Bz_6 and C_Bz_7), 135.3 and 139.1 (C_Bz_2 and C_Bz_4), 140.8 and 143.7 (C_F_4 and C_F_9), 153.4 (C_F_1), 166.2 (C_Bz_1), 178.2 (C(O)_Ac_), 178.6 (C_βA_1) ppm; ^195^Pt NMR ((CD_3_)_2_SO) *δ*: 1214 ppm.

#### 3.2.4. Synthesis of (*OC*-6-44)-Acetato-[7-(3-(fmoc-amino)-benzamido)-heptanoato]-diammine-dichlorido-platinum(IV), **7**

In a 10 mL round-bottom flask, 0.100 g of **5** (0.179 mmoL), 0.086 g of 3-(Fmoc-amino)benzoic acid (0.228 mmoL), and 0.136 g of the coupling agent HATU (0.358 mmoL) were suspended in 3 mL of anhydrous DMF. Then, 50 µL of DIPEA (0.286 mmoL) was added to the yellowish suspension, which turned to orange. The reaction mixture was left to be stirred overnight at room temperature in the dark. The solvent was then removed under reduced pressure, obtaining an orange oil that was recrystallized with dichloromethane and diethylether until the precipitation of a yellow-brown solid. Then, this solid residue was washed four times with diethylether, five times with 1% *w*/*w* formic acid to remove DIPEA, and twice with cold ultrapure water. Finally, the resulting powder was dried under a gentle nitrogen flow, obtaining 0.062 g of yellowish powder (yield 74%). Anal. calcd. for C_31_H_38_Cl_2_N_4_O_7_Pt: C, 44.08; H, 4.53; N, 6.63; found: C, 43.73; H, 4.30; N, 6.91. ESI-MS (positive ion mode): 845 *m*/*z*; calcd. for C_31_H_39_Cl_2_N_4_O_7_Pt [M + H]^+^ 845 *m*/*z*. ^1^H NMR ((CD_3_)_2_SO) *δ*: 1.28 (m, 4H, H_AH_4 and H_AH_5), 1.48 (m, 4H, H_AH_3 and H_AH_6), 1.90 (s, 3H, CH_3-Ac_), 2.20 (m, 2H, H_AH_2), 3.21 (m, 2H, H_AH_7), 4.31 (m, 1H, H_F_3), 4.47 (m, 2H, H_F_2), 6.52 (m, 6H, NH_3_), 7.35 (m, 2H, H_F_6), 7.42 (m, 2H, H_F_7), 7.75 (m, 2H, H_F_5), 7.90 (m, 2H, H_F_8), 7.25–8.00 (m, 4H, H_Bz_3, H_Bz_5, H_Bz_6, and H_Bz_7), 8.36 (m, 1H, NH_HA_), 9.83 (s, 1H, NH_Bz_) ppm; ^13^C NMR ((CD_3_)_2_SO) *δ*: 22.9 (CH_3-Ac_), 25.4 (C_AH_3), 26.3 (C_AH_5), 28.3 (C_AH_4), 29.0 (C_AH_6), 35.7 (C_AH_2), 39.5 (C_AH_7, under (CD_3_)_2_SO), 46.6 (C_F_3), 65.7 (C_F_2), 117.9 (C_Bz_3), 120.2 (C_F_8), 120.5 and 120.9 (C_Bz_5 and C_Bz_7), 125.1 (C_F_5), 127.1 and 127.9 (C_F_6 and C_F_7), 128.6 (C_Bz_6), 135.6 (C_Bz_2), 138.9 (C_Bz_4), 140.8 and 143.7 (C_F_4 and C_F_9), 153.4 (C_F_1), 166.1 (C_Bz_1), 178.2 (C(O)_Ac_), 180.9 (C_AH_1) ppm; ^195^Pt NMR ((CD_3_)_2_SO) *δ*: 1222 ppm.

#### 3.2.5. Synthesis of (*OC*-6-44)-Acetato[3-(3-aminobenzamido)-propanoato]-diammine-dichlorido-platinum(IV), **8**

In a 10 mL round-bottom flask, 0.100 g of **6** (0.127 mmoL) was added to 3 mL of a 6.7% solution of piperidine in DMF (200 µL in 2.8 mL), obtaining an orange solution. After 1 h of stirring at room temperature in the dark, the excess solvent was removed under reduced pressure. The resulting yellow solid was initially washed with a few drops of acetone and 2 mL of diethylether; then, it was washed four times with diethylether, four times with chloroform, and once again with diethylether. The solid residue was dried under a gentle nitrogen flow, obtaining 0.054 g of brown powder (yield 75%). Anal. calcd. for C_12_H_20_Cl_2_N_4_O_5_Pt: C, 25.45; H, 3.56; N, 9.89; found: C, 25.67; H, 3.65; N, 9.67. ESI-MS (positive ion mode): 567 *m*/*z*; calcd. for C_12_H_21_Cl_2_N_4_O_5_Pt [M + H]^+^ 567 *m*/*z*. ^1^H NMR ((CD_3_)_2_SO) *δ*: 1.92 (s, 3H, CH_3-Ac_), 2.44 (m, 2H, H_βA_2 or H_βA_3), 3.40 (m, 2H, H_βA_2 or H_βA_3), 5.18 (s, 2H, NH_2_-_Bz_), 6.53 (m, 6H, NH_3_), 6.67 (d, *J*^3^ = 7.8 Hz, 1H, H_Bz_5), 6.90 (d, *J*^3^ = 7.8 Hz, 1H, H_Bz_7), 7.01 (s, 1H, H_Bz_3), 7.03 (t, *J*^3^ = 7.8 Hz, 1H, H_Bz_6) ppm; ^13^C NMR ((CD_3_)_2_SO) *δ*: 22.8 (CH_3-Ac_), 36.0 and 36.1 (C_βA_2 and C_βA_3), 112.7 (C_Bz_3), 114.3 (C_Bz_7), 116.3 (C_Bz_5), 128.5 (C_Bz_6), 135.4 (C_Bz_2), 148.5 (C_Bz_4), 166.9 (C_Bz_1), 178.2 (C(O)_Ac_), 178.7 (C_βA_1) ppm; ^195^Pt NMR ((CD_3_)_2_SO) *δ*: 1214 ppm.

#### 3.2.6. Synthesis of (*OC*-6-44)-Acetato-[7-(3-aminobenzamido)-heptanoato]-diammine-dichlorido-platinum(IV), **9**

In a 10 mL round-bottom flask, 0.100 g of **7** (0.118 mmoL) was added to 3 mL of a 6.7% solution of piperidine in DMF (200 µL in 2.8 mL), obtaining an orange solution. After 1 h of stirring at room temperature in the dark, the excess solvent was removed under reduced pressure, obtaining a yellow solid that was initially washed with a few drops of acetone and 2 mL of diethylether and then with diethylether (four times), with chloroform (four times), and once again with diethylether. Finally, the solid was dried under a gentle nitrogen flow, obtaining 0.070 g of brown powder (yield 96%). Anal. calcd. for C_16_H_28_Cl_2_N_4_O_5_Pt: C, 30.88; H, 4.53; N, 9.00; found: C, 30.67; H, 4.78; N, 8.79. ESI-MS (positive ion mode): 623 *m*/*z*; calcd for C_16_H_29_Cl_2_N_4_O_5_Pt [M + H]^+^ 623 *m*/*z*. ^1^H NMR ((CD_3_)_2_SO) *δ*: 1.27 (m, 4H, H_HA_4 and H_HA_5), 1.47 (m, 4H, H_HA_3 and H_HA_6), 1.90 (s, 3H, CH_3-Ac_), 2.20 (m, 2H, H_HA_2), 3.19 (m, 2H, H_HA_7), 5.18 (s, 2H, NH_2-Bz_), 6.53 (m, 6H, NH_3_), 6.66 (dd, *J*^3^ = 7.8 Hz, *J*^4^ = 1.6 Hz, 1H, H_Bz_5), 6.91 (dd, *J*^3^ = 7.8 Hz, *J*^4^ = 1.6 Hz, 1H, H_Bz_7), 6.99 (s, 1H, H_Bz_3), 7.05 (t, *J*^3^ = 7.8 Hz, 1H, H_Bz_6), 8.16 (m, 1H, NH_HA_) ppm; ^13^C NMR ((CD_3_)_2_SO) *δ*: 22.9 (CH_3-Ac_), 25.4 (C_HA_3), 26.3 (C_HA_5), 28.3 (C_HA_4), 29.0 (C_HA_6), 35.9 (C_HA_2), 39.5 (C_HA_7 under (CD_3_)_2_SO), 112.7 (C_Bz_5), 114.2 (C_Bz_7), 116.1 (C_Bz_3), 128.5 (C_Bz_6), 135.7 (C_Bz_2), 148.5 (C_Bz_4), 166.8 (C_Bz_1), 178.1 (C(O)_Ac_), 180.8 (C_HA_1) ppm; ^195^Pt NMR ((CD_3_)_2_SO) *δ*: 1223 ppm.

### 3.3. Reduction Studies

The reduction of complexes with ascorbic acid was followed by monitoring of the reduction of the area of their chromatographic peaks via RP-HPLC over 24 h at 37 °C. The Pt compounds were dissolved in methanol or DMF (up to 20 mM) and diluted with different quantities of 50 mM 4-(2-hydroxyethyl)piperazine-1-ethanesulfonic acid (HEPES) buffer (pH = 7.5) containing 10 mM ascorbic acid. The mixtures were microfiltered to eliminate the precipitated complex. The chromatographic conditions were similar to those for the determination of lipophilicity (see Section 3.4), with different percentages of methanol depending on the complex.

### 3.4. Lipophilicity

RP-HPLC was used to evaluate the capacity factors of the compounds as reported elsewhere [43,44,52]. Briefly, a chromatogram for each Pt complex (0.5 mM) was run on a C18 column Phenosphere-NEXT (5-μm, 250 × 4.6 mm ID) with an eluant composition of 15 mM formic acid/CH_3_OH 30/70 and a flow rate of 0.5 mL min^−1^. The corresponding retention time *t*_R_ was used to calculate log *k*′, where *k*′ = (*t*_R_ − *t*_0_)/*t*_0_. The dead time of the column *t*_0_ was the *t*_R_ of KCl, added as unretained internal reference.

### 3.5. Biological Procedures

Four MPM cell lines were used in this work: MSTO-211H (ICLC HL01018) was obtained from the Istituto Scientifico Tumori cell-bank (IST Genoa, Italy), REN was kindly provided by S. M. Albelda (University of Pennsylvania, Philadelphia, PA, USA), and MM98 and BR95 were derived from pleural effusions of untreated MPM patients [53]. Mycoplasma infection was excluded using Mycoplasma PlusTM PCR Primer Set kit from Stratagene (La Jolla, CA, USA). The polyclonal antibodies specific for poly(ADP-ribose) polymerase 1 (PARP-1), tubulin, and HSC70 were purchased from Santa Cruz Biotechnology (Santa Cruz CA, USA). The monoclonal antibody specific for poly(ADP–ribose) was from Alexis (Vinci, FI, Italy). Anti-mouse and anti-rabbit IgG peroxidase conjugated antibodies and chemical reagents were from Sigma-Aldrich (St Louis, MO, USA). ECL, nitrocellulose membranes, and protein assay kit were from Bio-Rad (Hercules, CA, USA). Culture media, sera, and antibiotics were from Thermo Fisher (Waltham, MA, USA).

#### 3.5.1. Cell Viability Assay

Cells were seeded at a density of 10 × 10^4^ cells per well in 6-well plates in RPMI medium supplemented with 10% fetal bovine serum (FBS), 100 μg mL^−1^ streptomycin, and 10 μg mL^−1^ penicillin and incubated overnight at 37 °C in a humidified environment containing 5% CO_2_ to allow adherence. Cisplatin was dissolved in 0.9% *w*/*v* aqueous NaCl brought to pH = 3 with HCl (final stock concentration 1 mM). The Pt(IV) complexes were dissolved in DMSO (final stock concentration 5 mM); the resulting solutions were microfiltered (0.45 μm) and their Pt content was checked by ICP-MS (see Section 3.5.2). These mother solutions were diluted in complete medium to the required concentration range. The DMSO content never exceeded 0.2% *v*/*v* (this concentration was found to be non-toxic to the cells tested).

Following 72 h of treatment with the diluent of the compound (Ctrl) or different doses of the Pt compounds, cells were trypsinized and stained with Trypan blue. The number of cells considered viable (unstained cells) was counted in a Bürker haemocytometer within 5 min after staining.

#### 3.5.2. Cell Uptake

MSTO-211H cells were seeded in 10 mm Petri dishes and challenged with the compounds and mixtures under investigations (10 μM) in complete medium. At the end of the exposure, cells were washed three times with phosphate buffered saline (PBS) and detached from the Petri dishes using 0.05% Trypsin 1X + 2% EDTA (HyClone, Thermo Fisher). The cells were harvested in fresh complete medium and an automatic cell counting device (Countess®, Life Technologies, Carlsbad, CA, USA) was used to measure the number and the mean diameter from every cell count. The harvested cells were transferred into a borosilicate glass tube and centrifuged at 1100× *g* rpm for 5 min at room temperature. The supernatant was carefully removed by aspiration, but about 200 μL of the supernatant was left in order to limit the cellular loss. Finally, the cellular pellets were stored at −80 °C. Platinum content determination was performed by ICP-MS (Thermo Optek X Series 2). Complete mineralization was obtained by the addition of 70% *w*/*w* HNO_3_ to each sample (after defrosting), followed by incubation for 1 h at 60 °C in an ultrasonic bath. Before the ICP-MS measurement, the HNO_3_ was diluted to a final 1% concentration. The instrumental settings were optimized to yield maximum sensitivity for platinum, and the most abundant isotopes of Pt (*m*/*z* = 195) and In (*m*/*z* = 115, used as internal standard) were quantified. The level of Pt found in cells after the treatment was normalized to the cell number and expressed as ng of Pt per 10^6^ cells [54].

#### 3.5.3. Multicellular Spheroids

Multicellular spheroids were generated in non-adsorbent round-bottomed 96-well plates, as previously described [55]. The 96-well plates were coated with a 1:24 dilution of polyHEMA (120 mg mL^−1^) in 95% ethanol and dried at 37 °C for 24 h. Before use, plates were sterilized by UV light for 30 min. For generation of multicellular spheroids, 1 × 10^4^ cells were added into each well of a polyHEMA-coated 96-well plate and placed in a 37 °C humidified incubator with 5% CO_2_. Every 24 h, 50% of supernatant was replaced with fresh medium.

#### 3.5.4. Cell Lysis and Immunoblot

For protein analysis, cells were extracted with 1% NP-40 lysis buffer (50 mM Tris-HCl pH 8.5 containing 1% NP-40, 150 mM NaCl, 10 mM EDTA, 10 mM NaF, 10 mM Na_4_P_2_O_7_, and 0.4 mM Na_3_VO_4_) with freshly added protease inhibitors (10 μg mL^−1^ leupeptin, 4 μg mL^−1^ pepstatin, and 0.1 U mL^−1^ aprotinin). Lysates were centrifuged at 13,000× *g* for 10 min at 4 °C and the supernatants were collected and assayed for protein concentration with the Bradford assay method (Bio-Rad). Proteins were separated by SDS-PAGE (sodium dodecyl sulfate polyacrylamide gel electrophoresis) under reducing conditions. Following SDS-PAGE, proteins were transferred to nitrocellulose, reacted with specific antibodies, and then detected with peroxidase-conjugate secondary antibodies and chemioluminescent ECL reagent. Digital images were taken with the Bio-Rad ChemiDocTM Touch Imaging System and quantified using Bio-Rad Image Lab 5.2.1.

#### 3.5.5. γH2AX Foci Immunofluorescence

MSTO-211H cells were plated on slides and treated for 72 h with diluent or 5 μM complex **6**. Slides were then fixed for 20 min with 4% paraformaldehyde (Sigma-Aldrich), rinsed with PBS, and then the cells were permeabilized for 5 min in 0.1% Triton X-100, followed by three 5 min PBS washes. Slides were treated thrice for 10 min in a blocking solution of 5% bovine serum albumin (BSA) (Invitrogen). Mouse anti-γH2AX antibody (Millipore) was added (1:100 in 1% BSA) and incubated for 1 h in a dark, humidified environment at room temperature. Slides were then exposed to a secondary goat anti-mouse antibody, FITC (1:100 in PBS) and incubated for 1 h in the dark, humidified environment at room temperature. Slides were rinsed thrice in PBS, mounted, and cell imaged with a Leica fluorescence microscope.

#### 3.5.6. Cellular Thermal Shift Assay (CETSA^®^)

For the intact cell experiments, MSTO-211H cells were treated with the diluent of the compound (Ctrl) or **6** (50 µM) in complete medium for 15 min at 37 °C, in a humidified environment containing 5% CO_2_. Cells were then washed, harvested, and diluted in PBS supplemented with complete protease inhibitors cocktail. Aliquots of the cell suspension (50 µL) were heated individually at different temperatures for 3 min in a dry heat block, followed by cooling for 3 min at room temperature. Cell suspensions were then freeze-thawed three times using liquid nitrogen. The soluble fractions (lysates) were separated from the cell debris by centrifugation at 13,000× *g* for 20 min at 4 °C. The supernatants were then analyzed by SDS-PAGE followed by Western blot analysis as previously described.

#### 3.5.7. RNA Isolation and Real-Time PCR

Total RNA was extracted using the guanidinium thiocyanate method. Starting from equal amounts of RNA, cDNA, used as a template for amplification in the real-time PCR (5 µg), was synthesized by the reverse transcription reaction using a RevertAid Minus First Strand cDNA Synthesis Kit from Fermentas-Thermo Scientific (Burlington, ON, Canada), using random hexamers as primers, according to the manufacturer’s instructions. Twenty nanograms of cDNA was used to perform RT-PCR amplification of mRNA.

## 4. Conclusions

Here we described the synthesis of four Pt(IV) complexes containing PARPis based on **3-ABA** at their axial positions.

All compounds reduced the viability of MPM cells with different histological subtypes, with IC_50_ values mostly in the micromolar range.

The mechanistic studies performed on MSTO-211H cells show that the leading PARPi-Pt(IV) conjugate **6** efficiently enters cells, binds, and inhibits PARP-1, also inducing apoptosis in 3D MCS.

The present study proves that the conjugation of PARPis to the Pt(IV) center to produce bifunctional complexes could be an applicable approach to develop new metal-based anticancer complexes as potential drug candidates for MPM therapy.

## Figures and Tables

**Table 1:** Lipophilicity (log *k*′), Pt uptake (ng Pt per 10^6^ MSTO-211H cells), and antiproliferative (IC_50_, μM) data for complexes **6**–**9** together with cisplatin, CDDP, and **SAA1**. IC_50_ data were obtained after 72 h of treatment of the malignant pleural mesothelioma cancer cell lines MSTO-211H (mixed or biphasic phenotype), REN (epithelioid), BR95 (epithelioid), and MM98 (sarcomatoid). Data are means ± standard deviation (sd) of at least three independent replicates. Numbers in parenthesis are the ratios IC_50_ (cisplatin)/IC_50_ (Pt(IV) complex).

Compound	Log *k*′	IC_50_ MSTO-211H (μM)	IC_50_ REN (μM)	IC_50_ BR95 (μM)	IC_50_ MM98 (μM)	Pt Uptake (ng Pt per 10^6^ Cells)
Cisplatin, CDDP	0.29	1.14 ± 0.07	3.07 ± 0.43	2.15 ± 0.29	1.75 ± 0.09	9.23 ± 0.96
**6**	4.22	0.69 ± 0.03 (1.6)	1.59 ± 0.07 (1.9)	0.83 ± 0.04 (2.6)	0.98 ± 0.03 (1.8)	10.64 ± 1.77
**8**	0.25	28.9 ± 2.9 (0.039)	46.9 ± 3.2 (0.065)	56.8 ± 4.3 (0.038)	58.4 ± 2.7 (0.030)	2.50 ± 1.16
**7**	5.84	1.48 ± 0.02 (0.77)	2.22 ± 0.08 (1.4)	2.19 ± 0.13 (0.98)	0.45 ± 0.08 (3.9)	10.86 ± 1.17
**9**	0.34	54.9 ± 3.1 (0.021)	38.9 ± 2.2 (0.079)	39.7 ± 2.7 (0.054)	44.1 ± 3.5 (0.049)	3.12 ± 0.78
**SAA1**	1.36	2.13 ± 0.05 (0.53)	5.73 ± 0.11 (0.53)	3.53 ± 0.06 (0.61)	1.96 ± 0.04 (0.89)	5.38 ± 1.80

## Data Availability

The data used to support the findings of this study are included within the article and the Supplementary Materials.

## Supplementary Materials

The following are available online, Figure S1: Sketch of the compounds under investigation and numbering scheme for NMR assignment, Figures S2–S7: ESI-MS and NMR characterization of **3**, Figures S8–S13: ESI-MS and NMR characterization of **5**, Figures S14–S19: ESI-MS and NMR characterization of **6**, Figures S20–S25: ESI-MS and NMR characterization of **7**, Figures S26–S31: ESI-MS and NMR characterization of **8**, Figures S32–S37: ESI-MS and NMR characterization of **9**.

## Sample Availability

Samples of the compounds are not available from the authors.
