# Supplementary material for: Unsymmetric Cisplatin-Based Pt(IV) Conjugates Containing a PARP-1 Inhibitor Pharmacophore Tested on Malignant Pleural Mesothelioma Cell Lines
Source: Molecules. 2021 Aug 5;26(16):4740. doi: 10.3390/molecules26164740 (PMC8402032; doi:10.3390/molecules26164740)
Supplement: Supplementary file 1 [file molecules-26-04740-s001.zip › molecules-1298327-supplementary.pdf]

# Unsymmetric cisplatin-based Pt(IV) conjugates containing a PARP-1 inhibitor pharmacophore tested on malignant pleural mesothelioma cell lines

Elisabetta Gabano[1], Giulia Pinton[2], Cecilia Balzano[1,2], Sara Boumya[2], Domenico Osella[1], Laura Moro[2,*], Mauro Ravera[1,*]

[1] Dipartimento di Scienze e Innovazione Tecnologica, Università del Piemonte Orientale, Viale Michel 11, 15121 Alessandria, Italy
[2] Dipartimento di Scienze del Farmaco, Università del Piemonte Orientale, Largo Donegani 2/3, 28100 Novara, Italy
* Correspondence: LM laura.moro@uniupo.it; MR mauro.ravera@uniupo.it

## SUPPORTING INFORMATION

**Table of content:**

**Figure S1**. Sketch of the compounds under investigation and numbering scheme for NMR assignment.

**Figure S2-Figure S7**. ESI-MS and NMR characterization of **3**.

**Figure S8-Figure S13**. ESI-MS and NMR characterization of **5**.

**Figure S14-Figure S19**. ESI-MS and NMR characterization of **6**.

**Figure S20-Figure S25**. ESI-MS and NMR characterization of **7**.

**Figure S26-Figure S31**. ESI-MS and NMR characterization of **8**.

**Figure S32-Figure S37**. ESI-MS and NMR characterization of **9**.

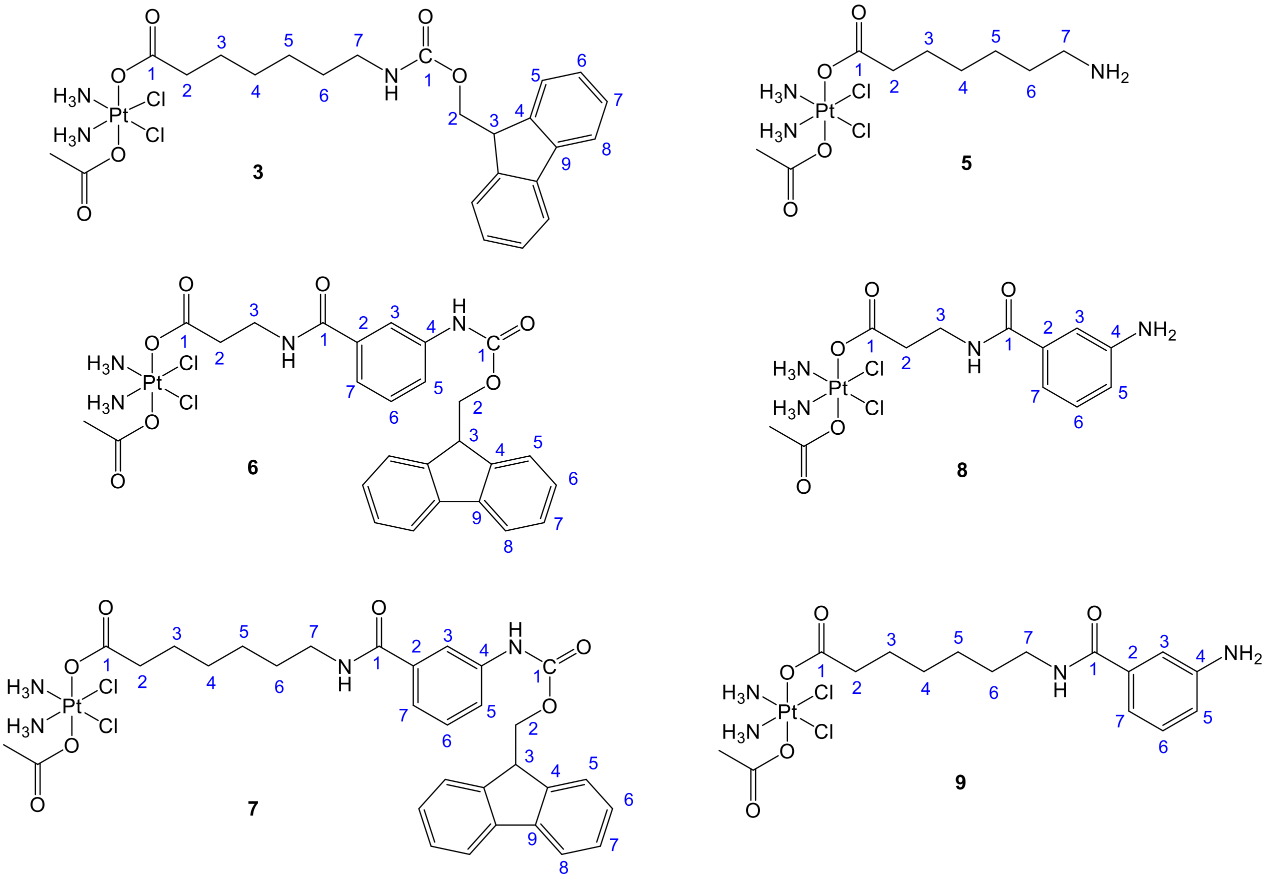


**Figure S1**. Sketch of the compounds under investigation and numbering scheme for NMR assignment. The assignment includes numbers and codes for the different portions of the molecules: Ac = acetate, AH = 7-aminoheptanoic acid, βA = β-alanine, F = Fmoc, Bz = 3-aminobenzoic acid.

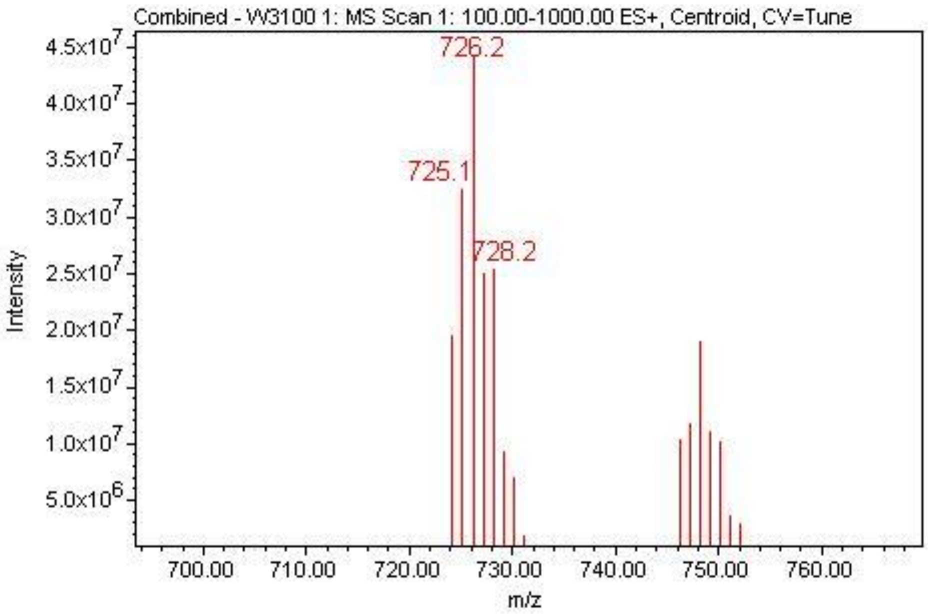


**Figure S2**. ESI-MS spectrum of complex **3**.

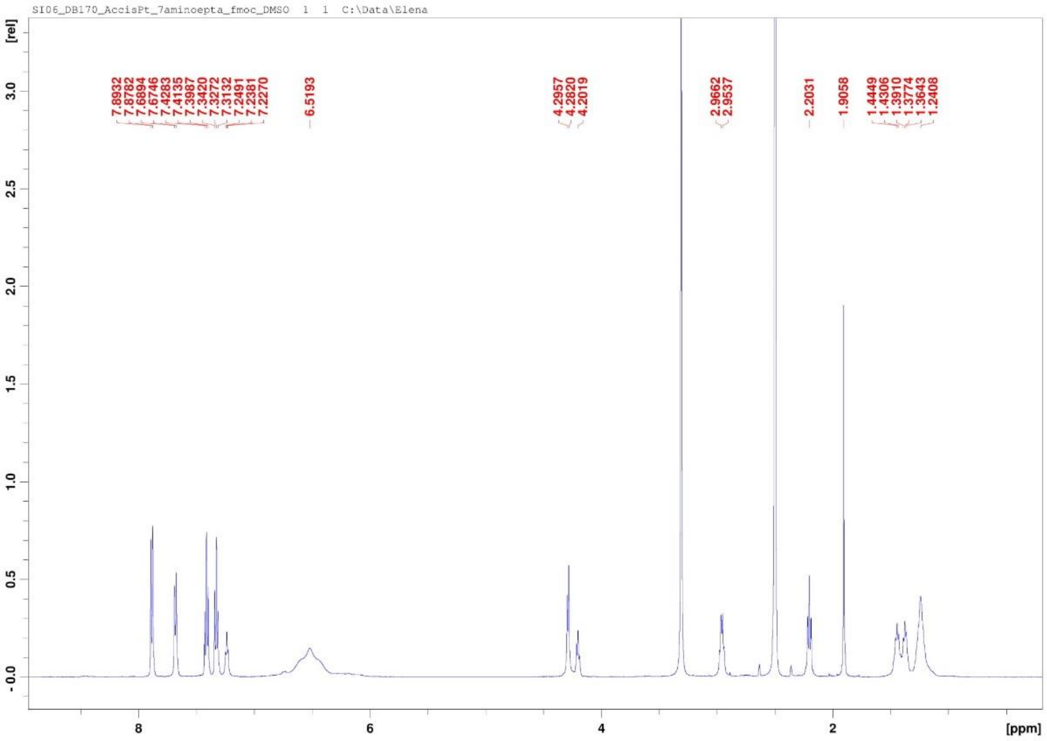


**Figure S3**. $^{1}$H NMR spectrum of complex **3**.

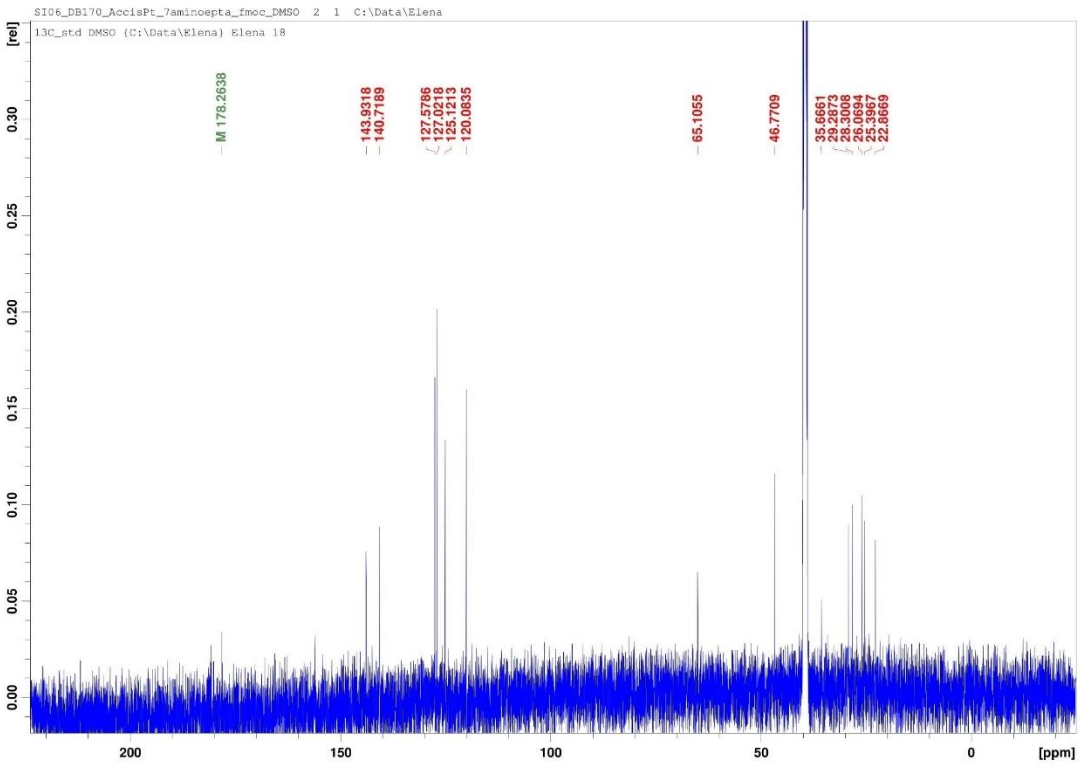


**Figure S4**. $^{13}$C NMR spectrum of complex **3**.

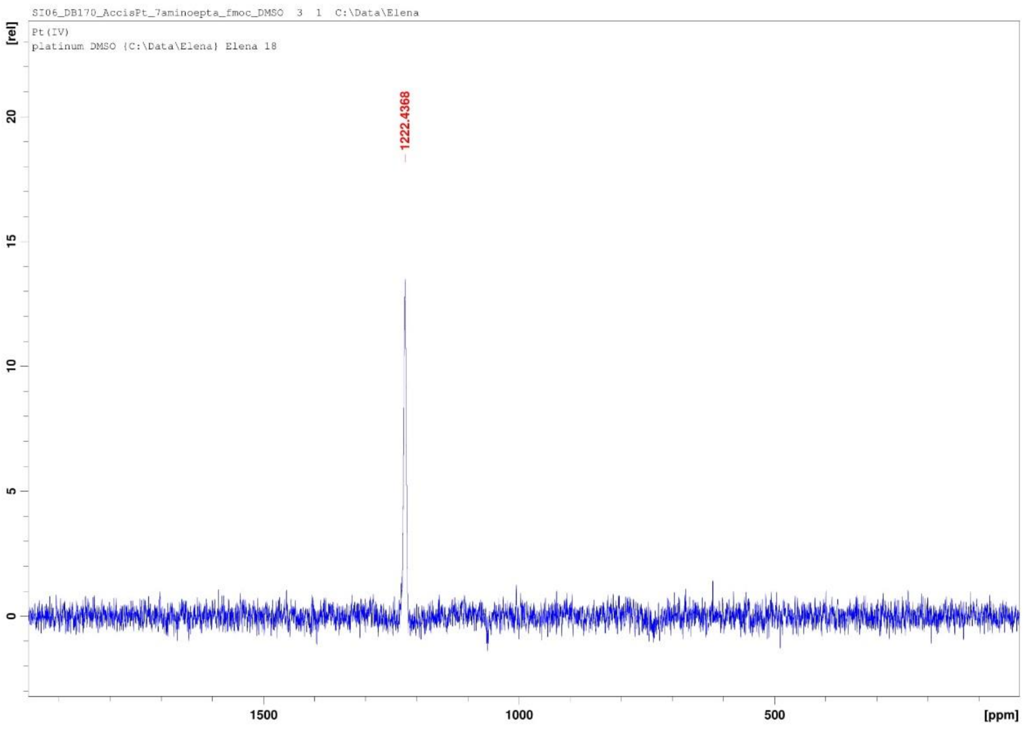


**Figure S5**. $^{195}$Pt NMR spectrum of complex **3**.

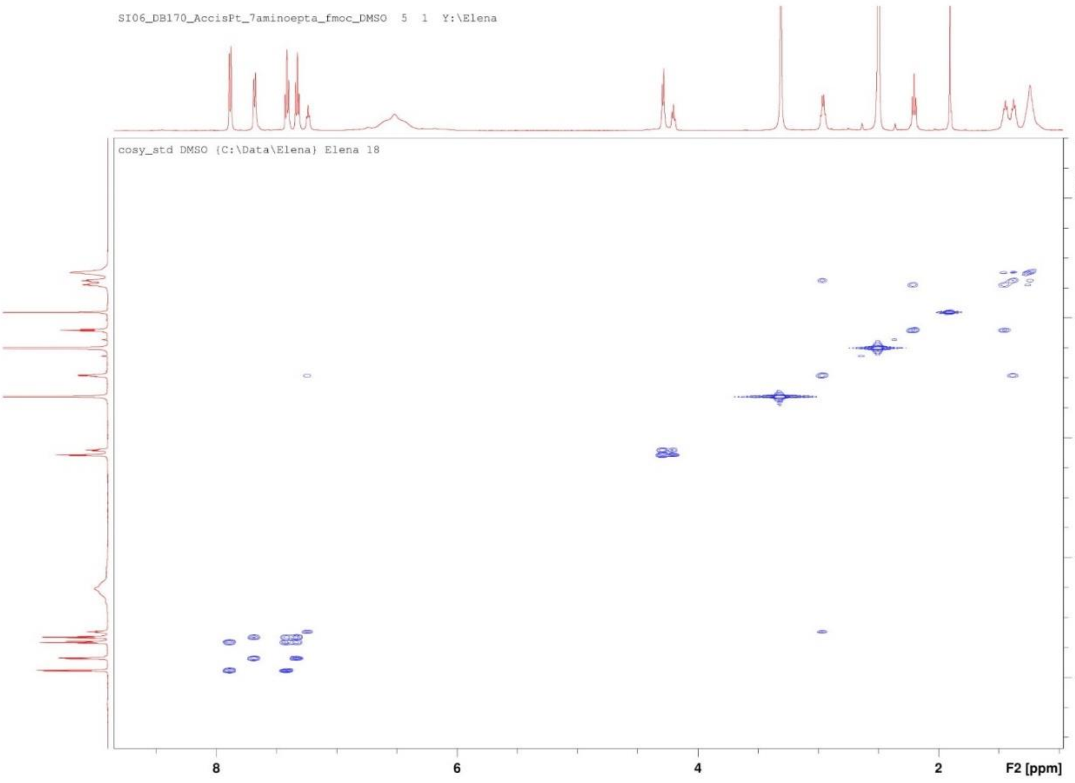


**Figure S6**. [$^1$H, $^1$H] COSY NMR spectrum of complex **3**.

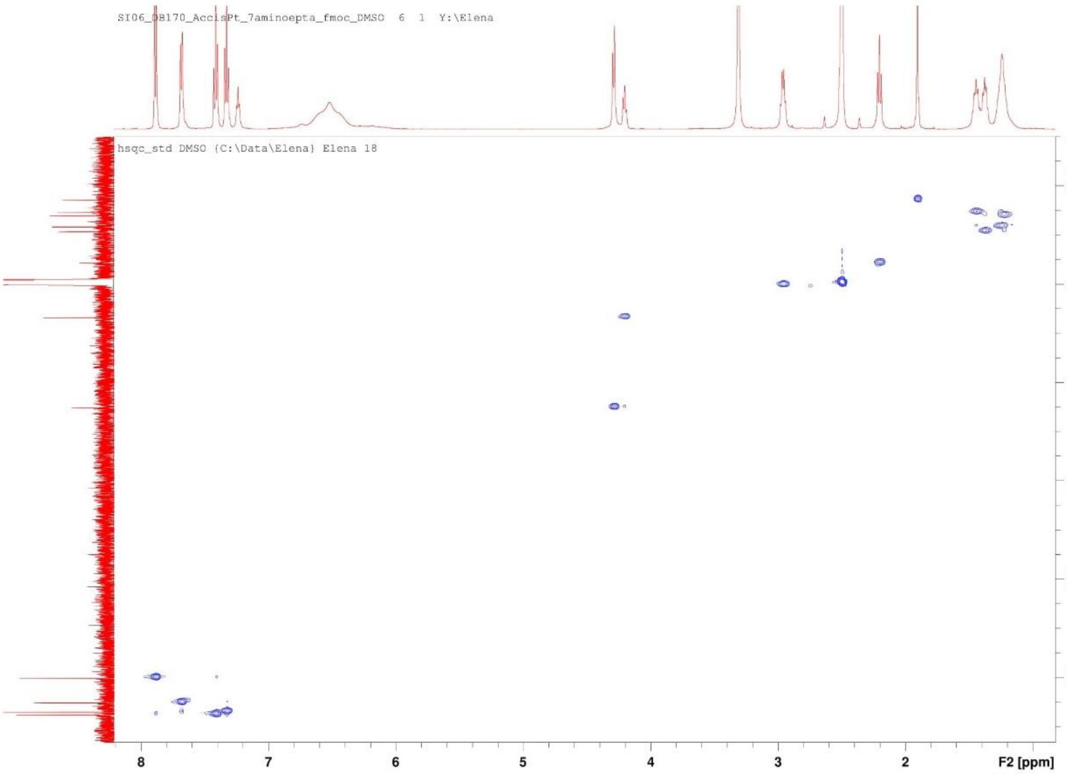


**Figure S7**. [$^1$H, $^{13}$C] HSQC NMR spectrum of complex **3**.

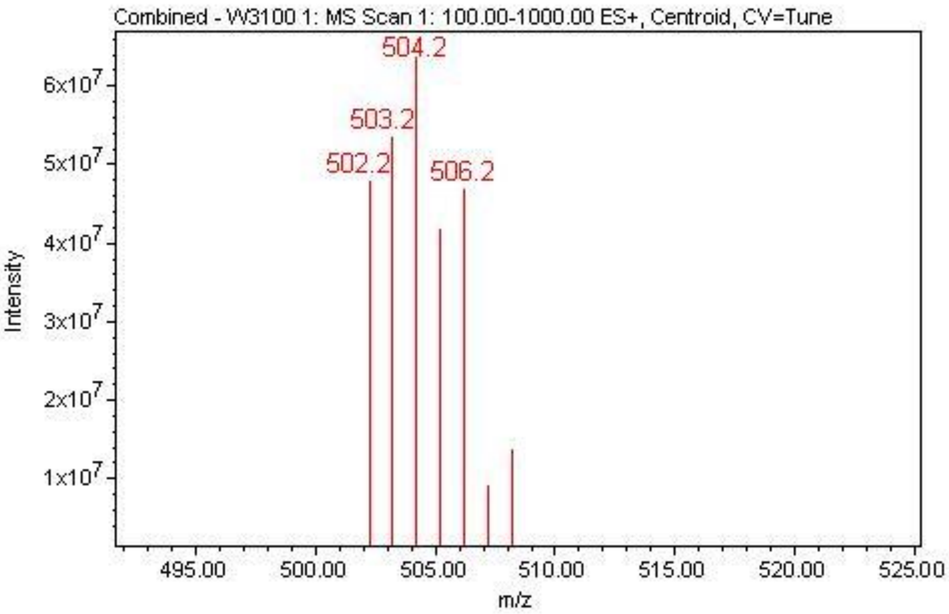


**Figure S8**. ESI-MS spectrum of complex **5**.

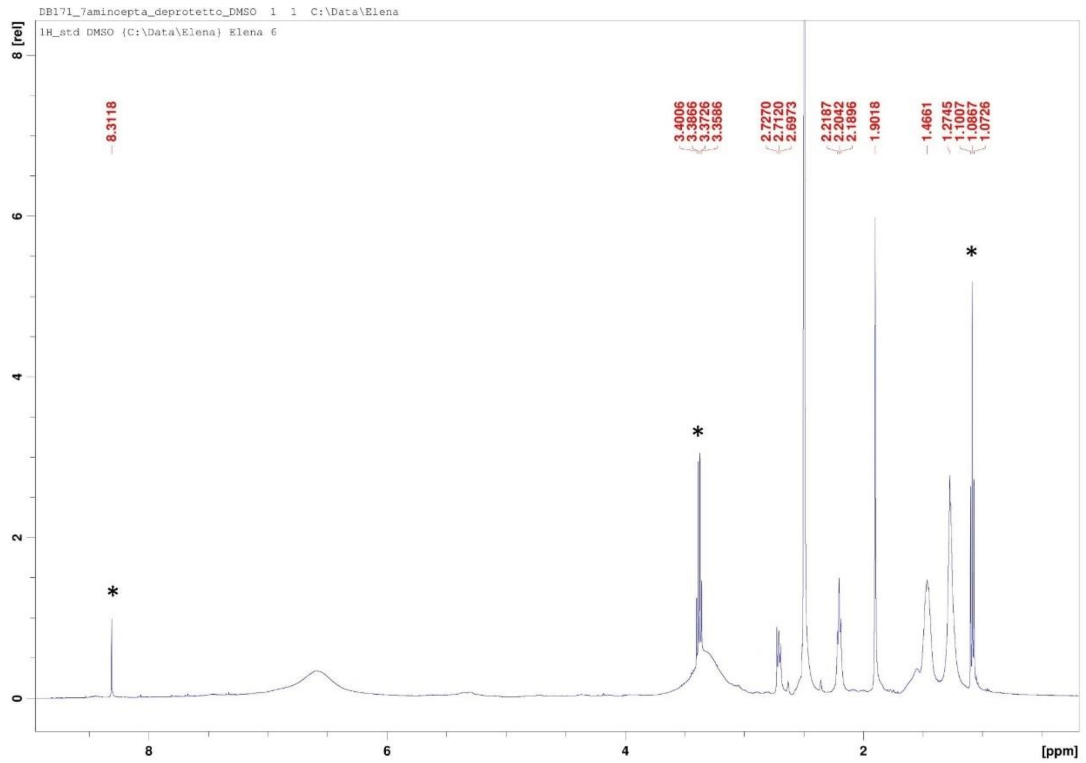


**Figure S9**. $^{1}$H NMR spectrum of complex **5**. The signals of resuidual diethyl ether and chloroform are also visible (*).

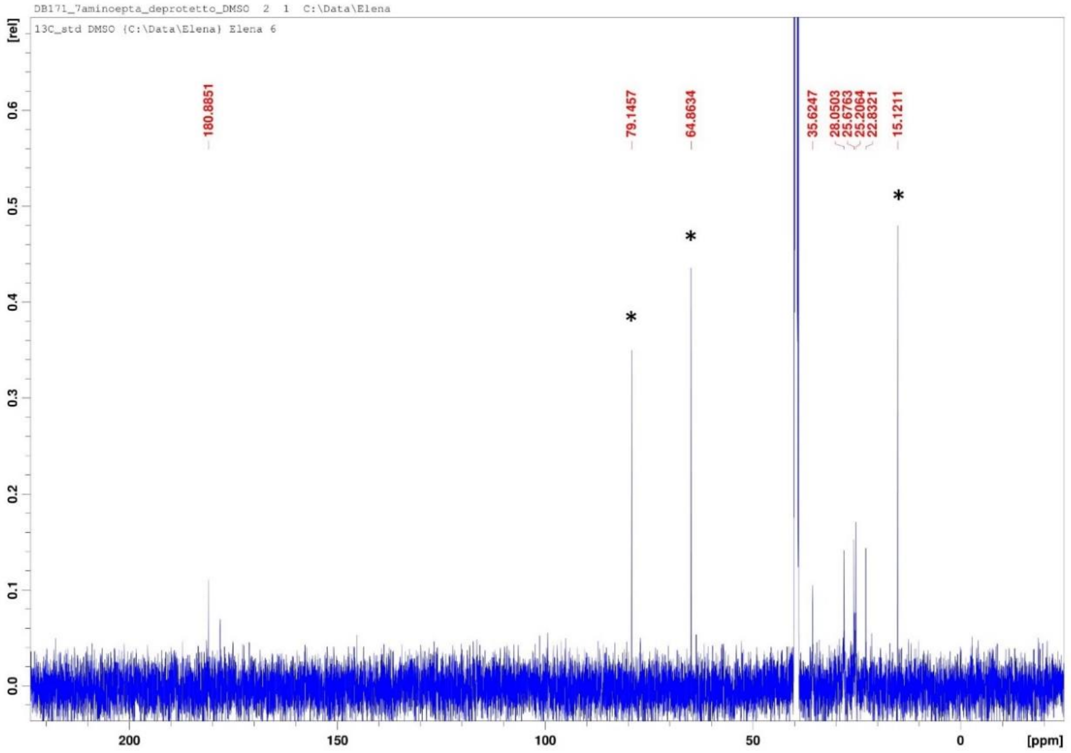


**Figure S10**. $^{13}$C NMR spectrum of complex **5**. The signals of resuidual diethyl ether and chloroform are also visible (*).

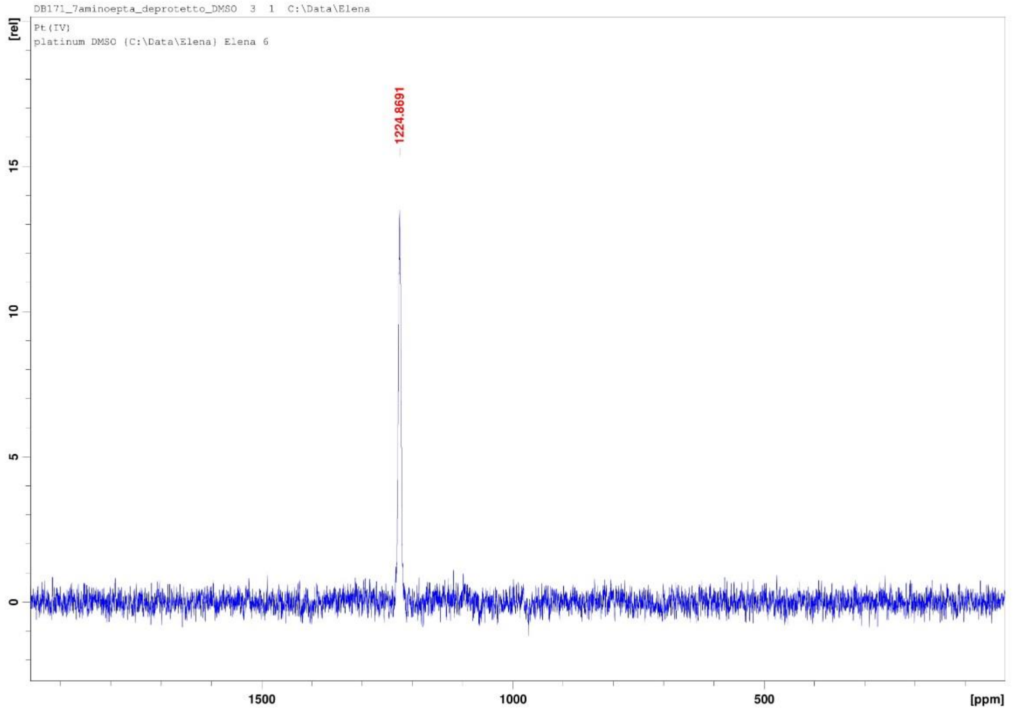


**Figure S11**. $^{195}$Pt NMR spectrum of complex **5**.

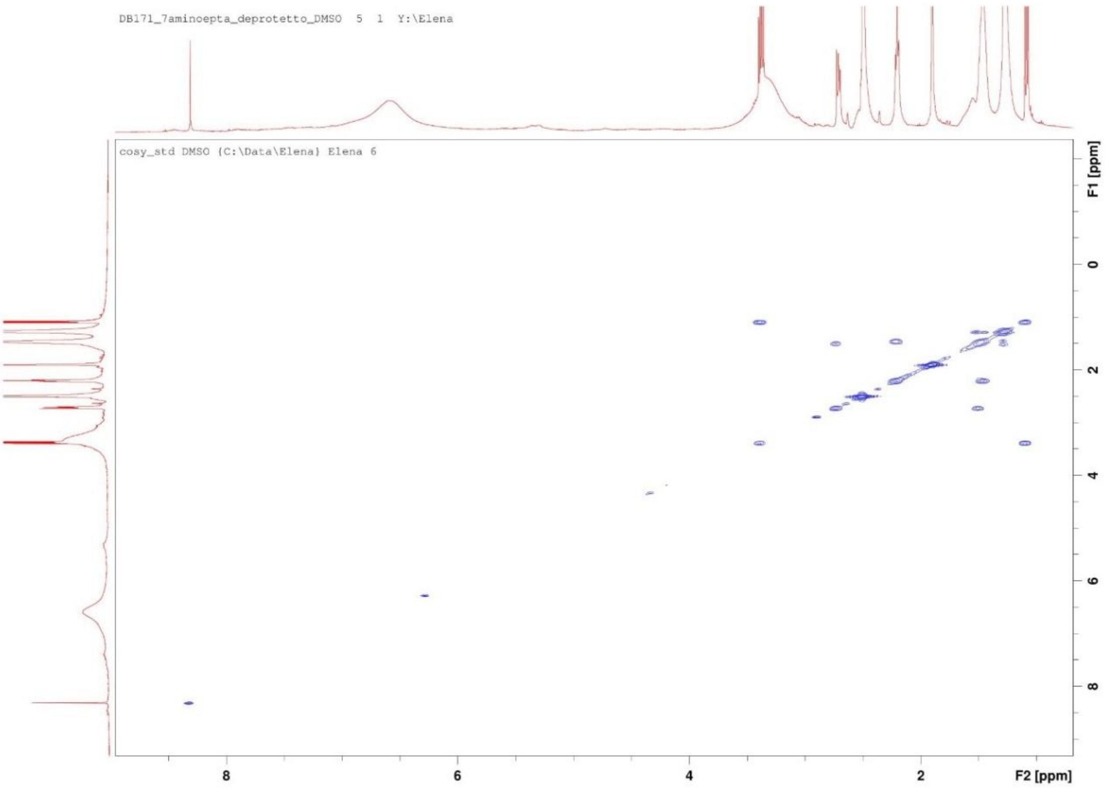


**Figure S12**. [$^{1}$H, $^{1}$H] COSY NMR spectrum of complex **5**.

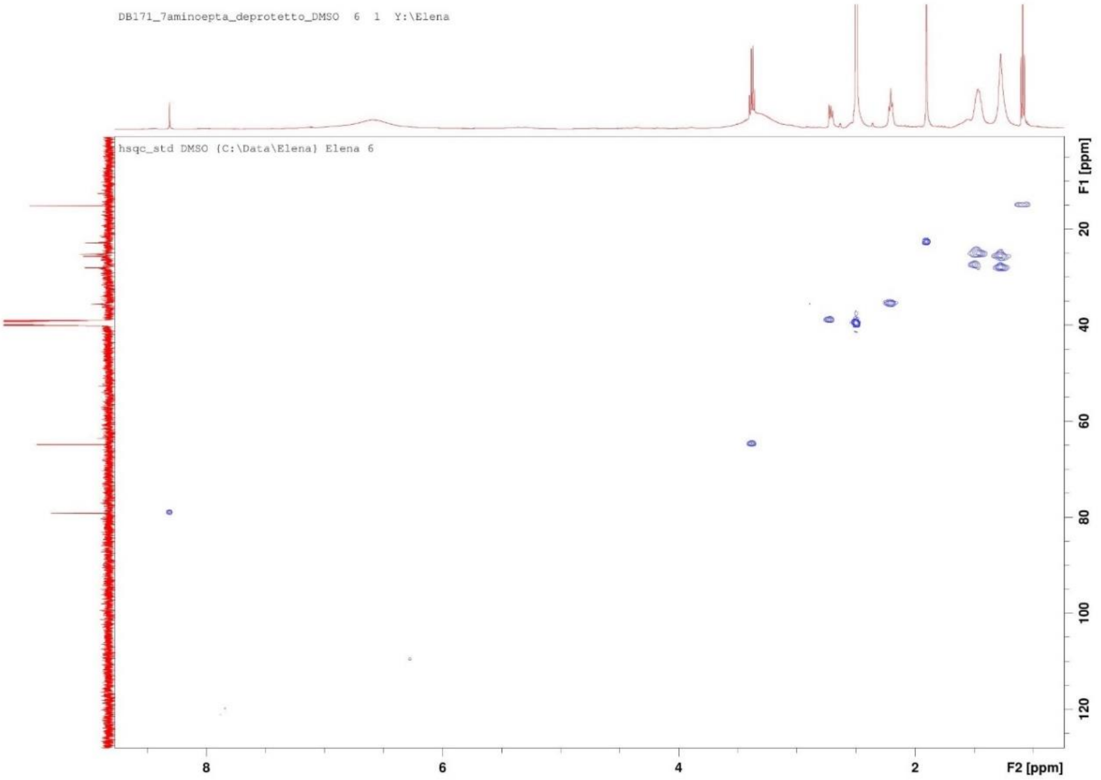


**Figure S13**. [$^{1}$H, $^{13}$C] HSQC NMR spectrum of complex **5**.

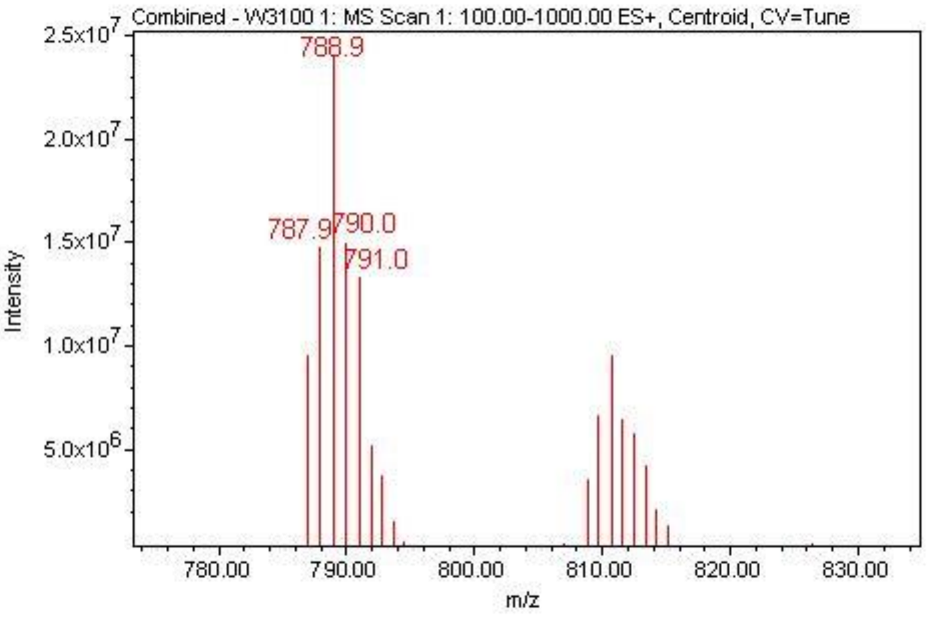


**Figure S14**. ESI-MS spectrum of complex **6**.

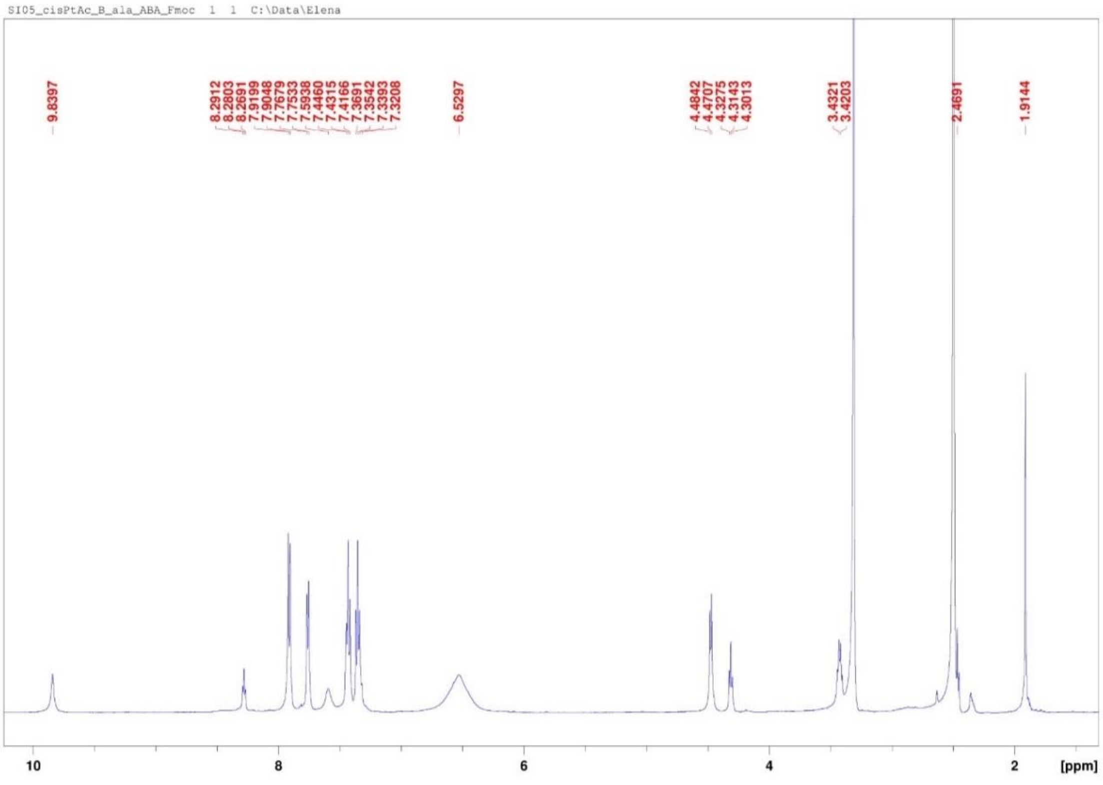


**Figure S15**. $^{1}$H NMR spectrum of complex **6**.

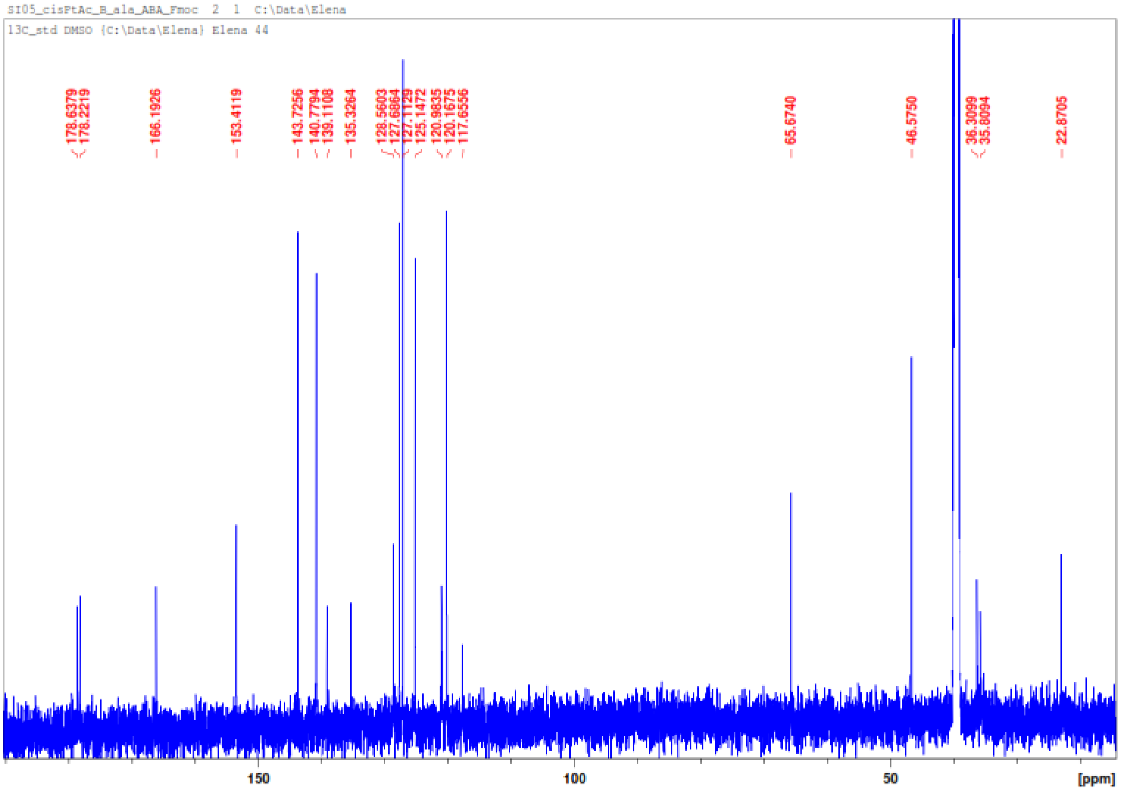


**Figure S16**. $^{13}C$ NMR spectrum of complex **6**.

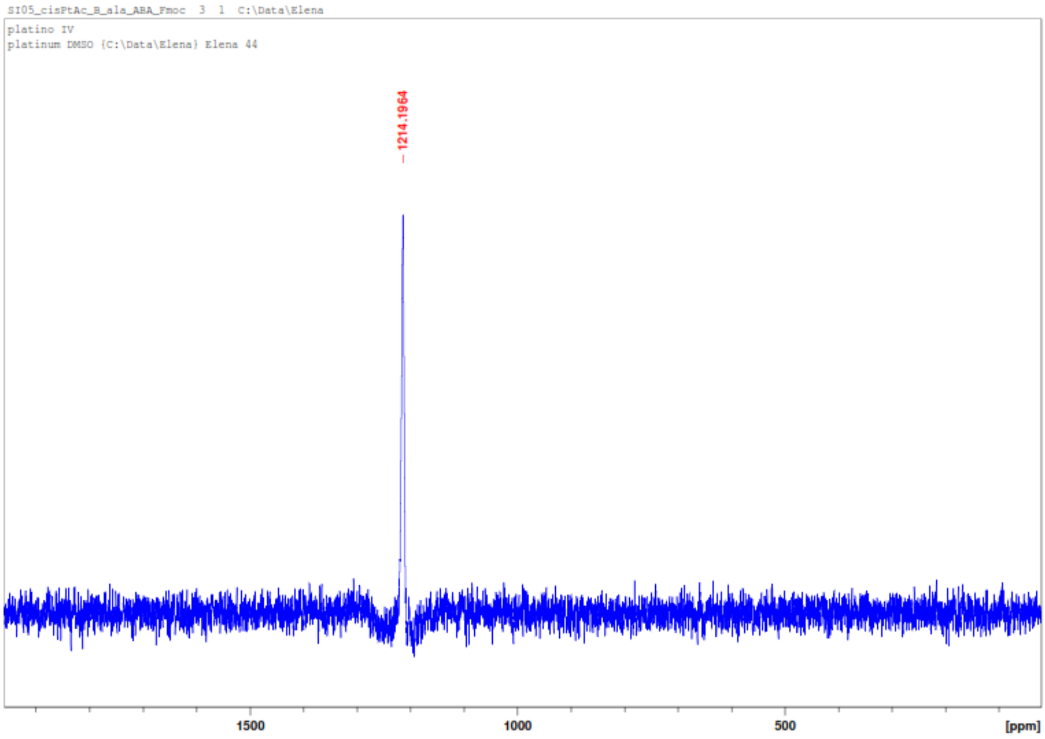


**Figure S17**. $^{195}Pt$ NMR spectrum of complex **6**.

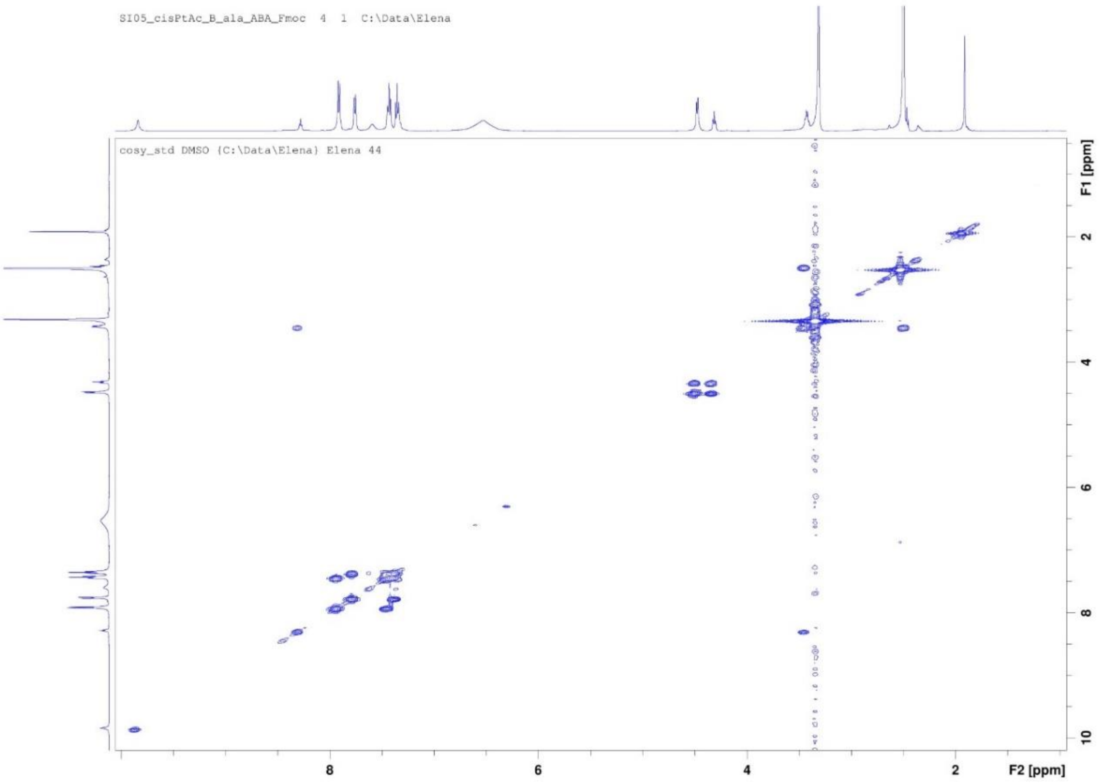


**Figure S18**. [$^1$H, $^1$H] COSY NMR spectrum of complex **6**.

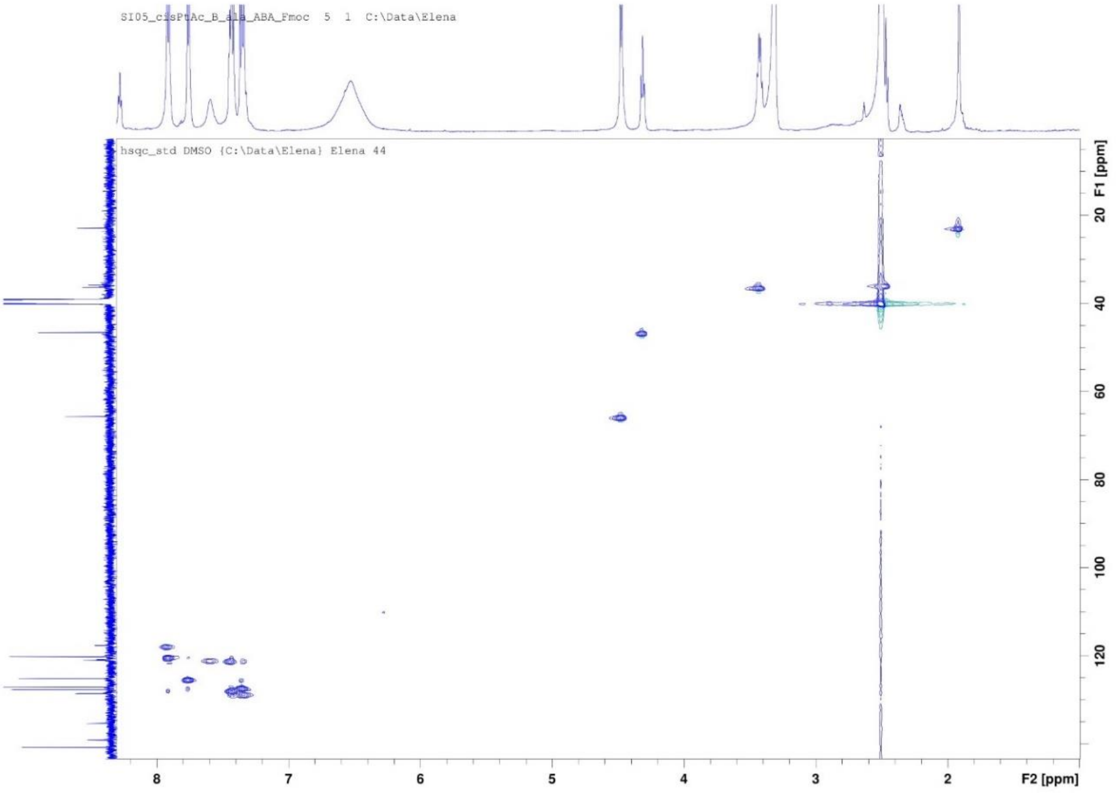


**Figure S19**. [$^1$H, $^{13}$C] HSQC NMR spectrum of complex **6**.

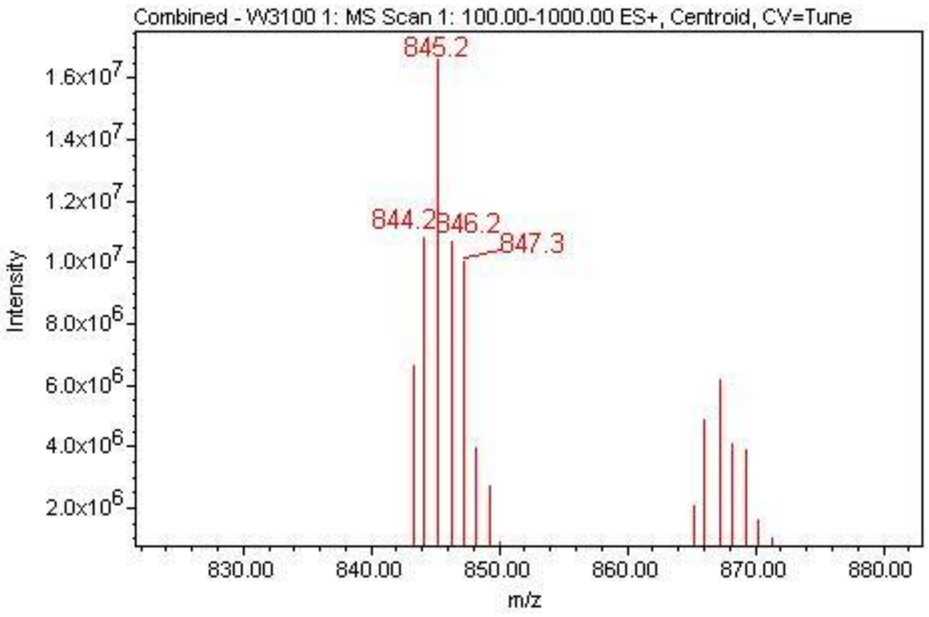


**Figure S20**. ESI-MS spectrum of complex **7**.

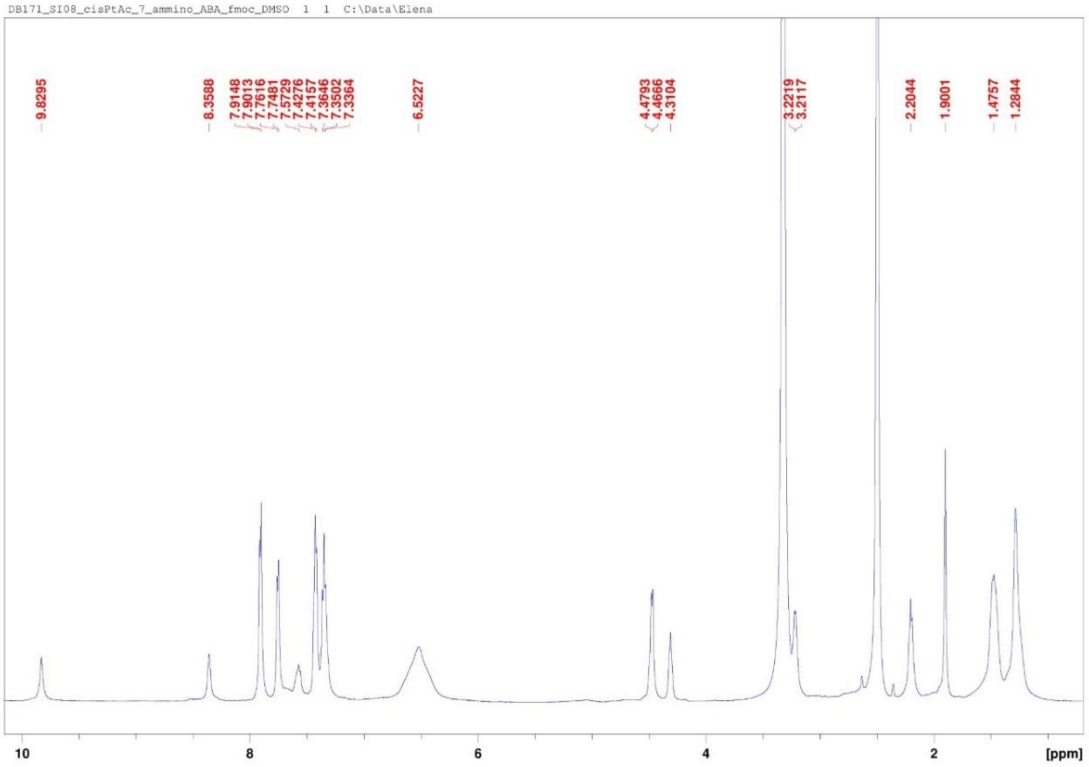


**Figure S21**. $^{1}$H NMR spectrum of complex **7**.

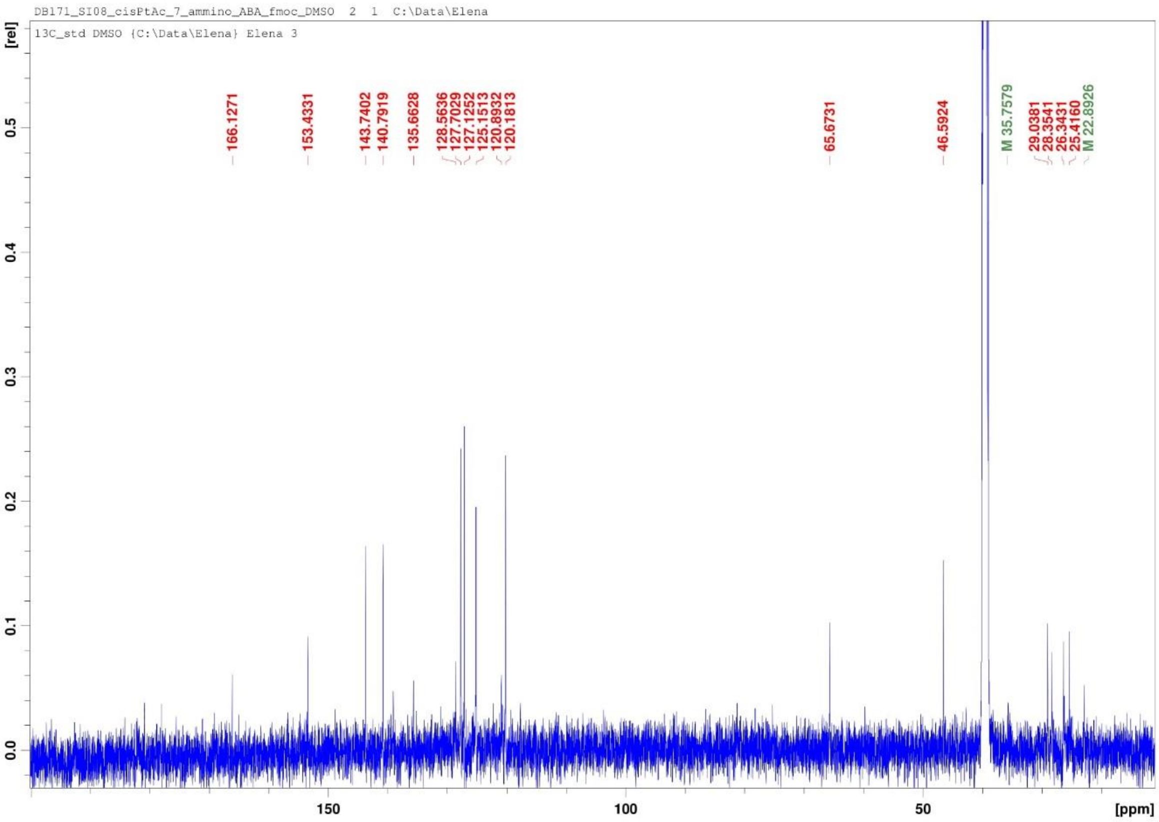


**Figure S22**. $^{13}$C NMR spectrum of complex **7**.

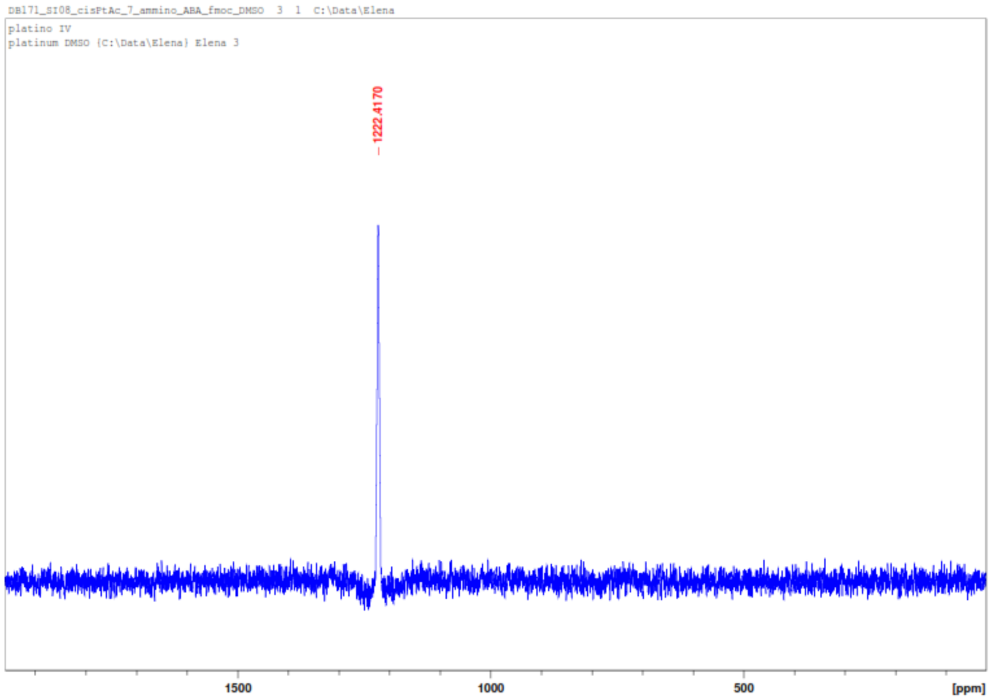


**Figure S23**. $^{195}$Pt NMR spectrum of complex **7**.

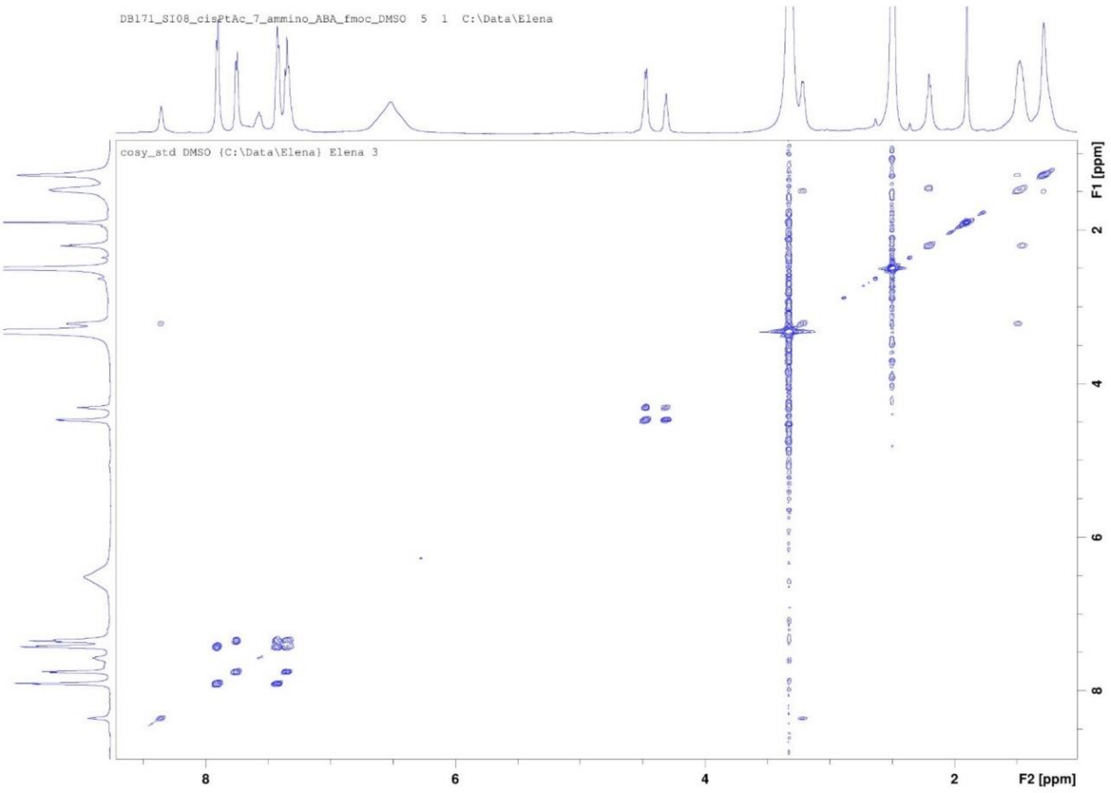


**Figure S24**. [$^{1}$H, $^{1}$H] COSY NMR spectrum of complex **7**.

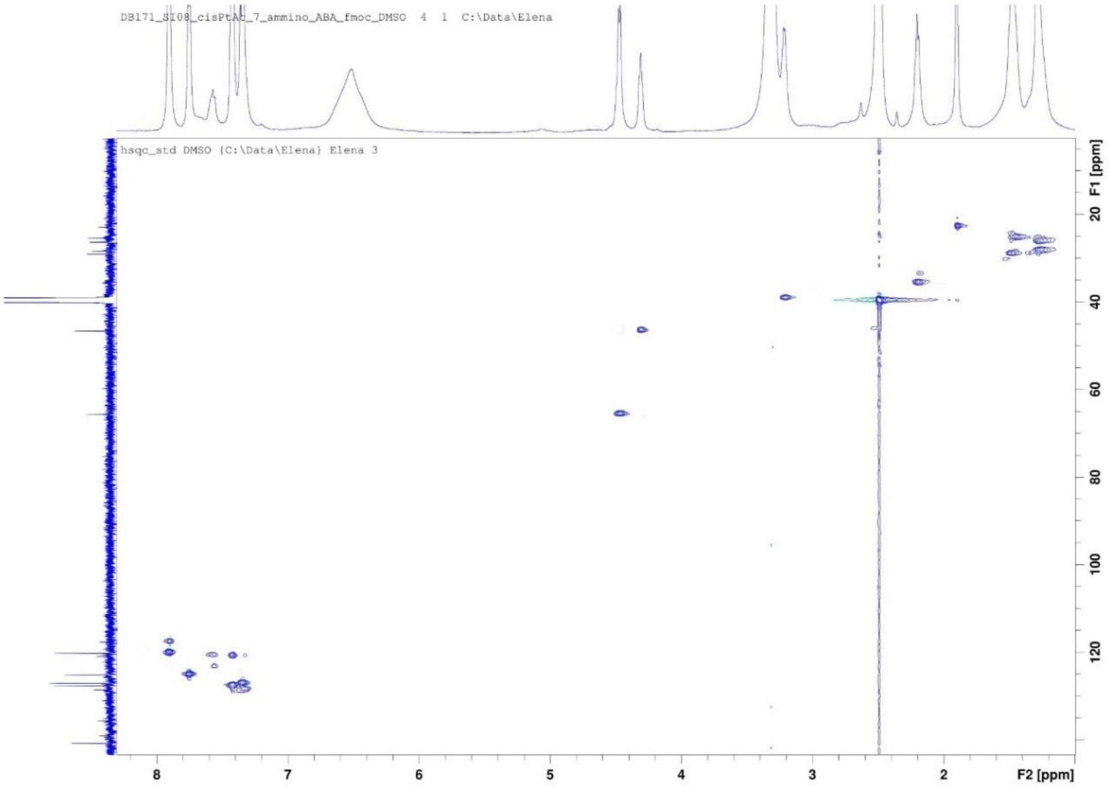


**Figure S25**. [$^{1}$H, $^{13}$C] HSQC NMR spectrum of complex **7**.

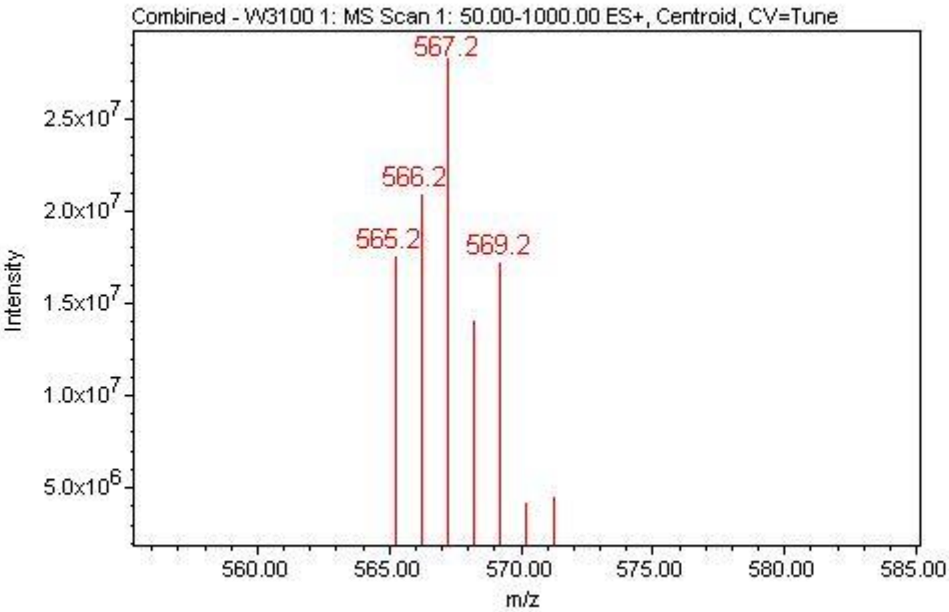


**Figure S26**. ESI-MS spectrum of complex **8**.

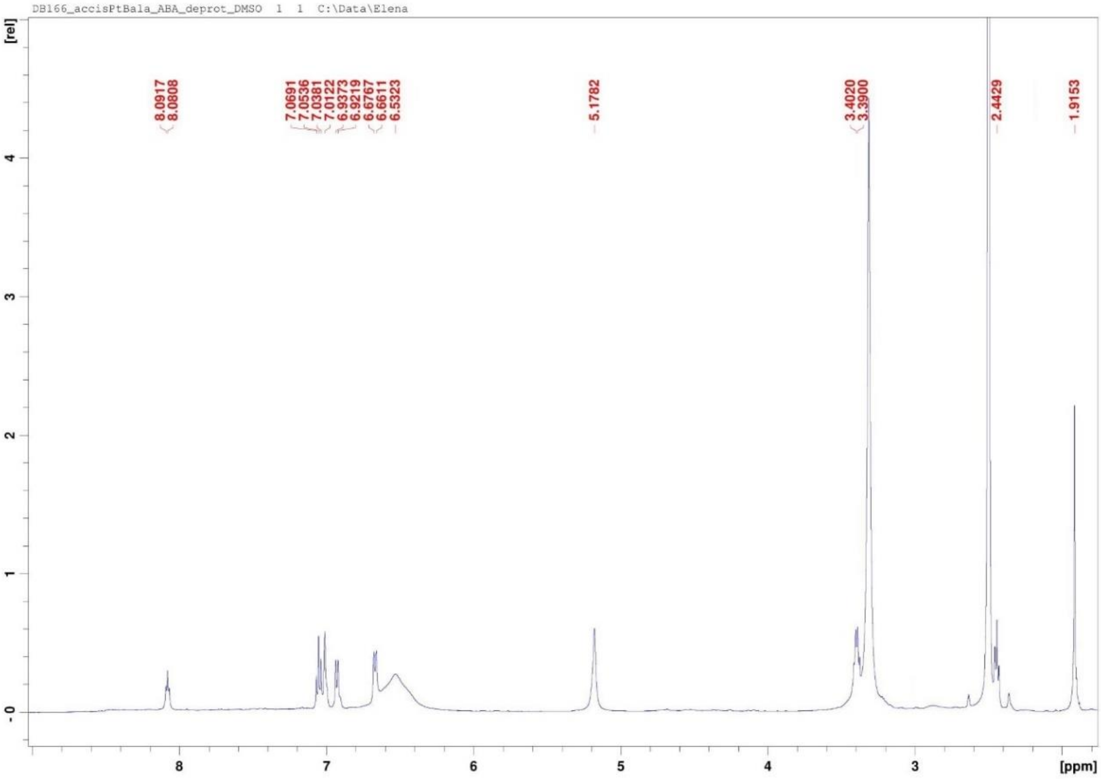


**Figure S27**. $^1$H NMR spectrum of complex **8**.

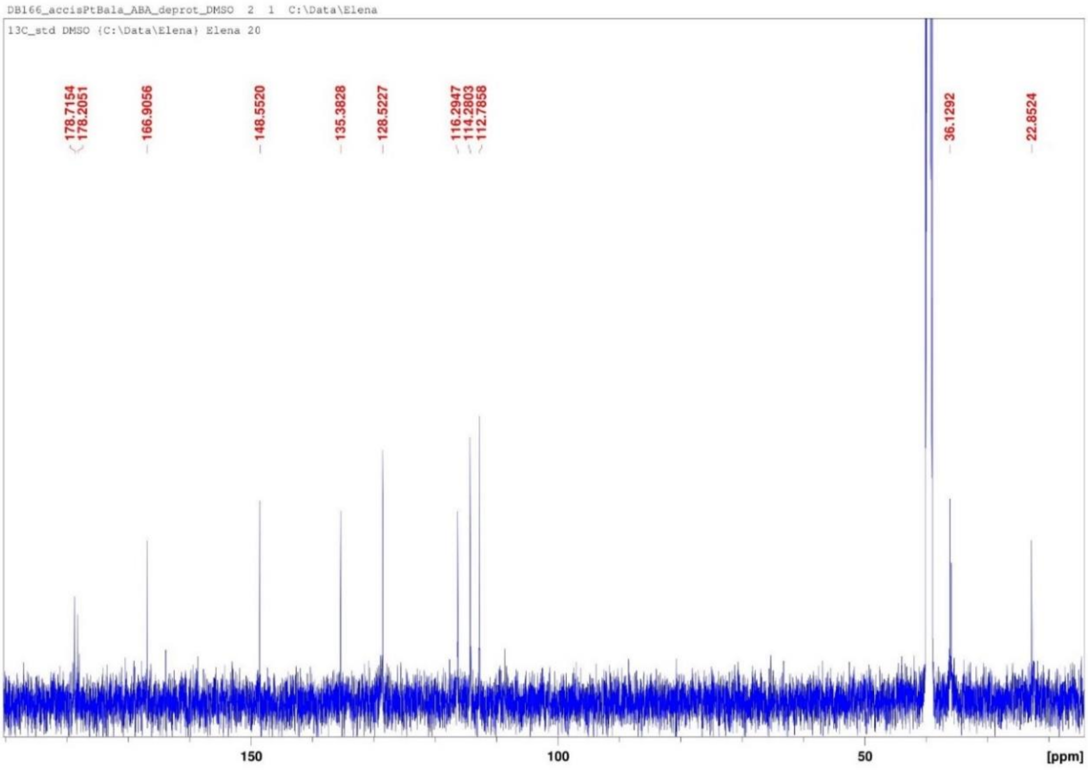


**Figure S28**. $^{13}$C NMR spectrum of complex **8**.

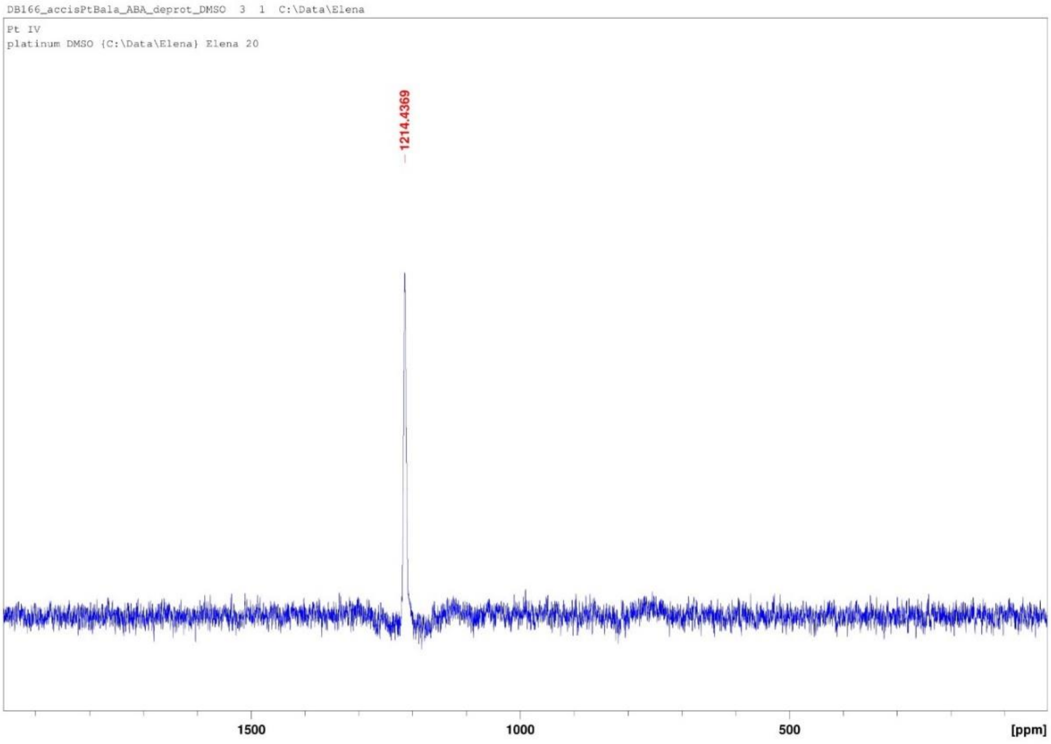


**Figure S29**. $^{195}$Pt NMR spectrum of complex **8**.

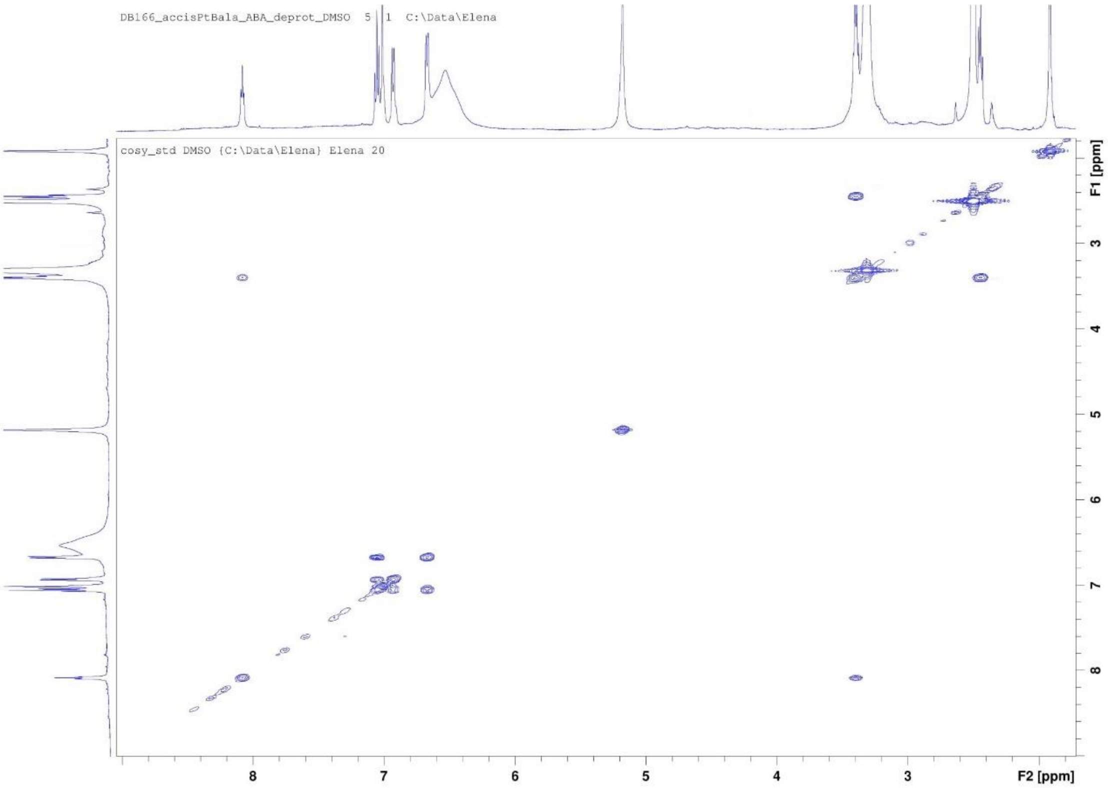


**Figure S30**. [$^{1}$H, $^{1}$H] COSY NMR spectrum of complex **8**.

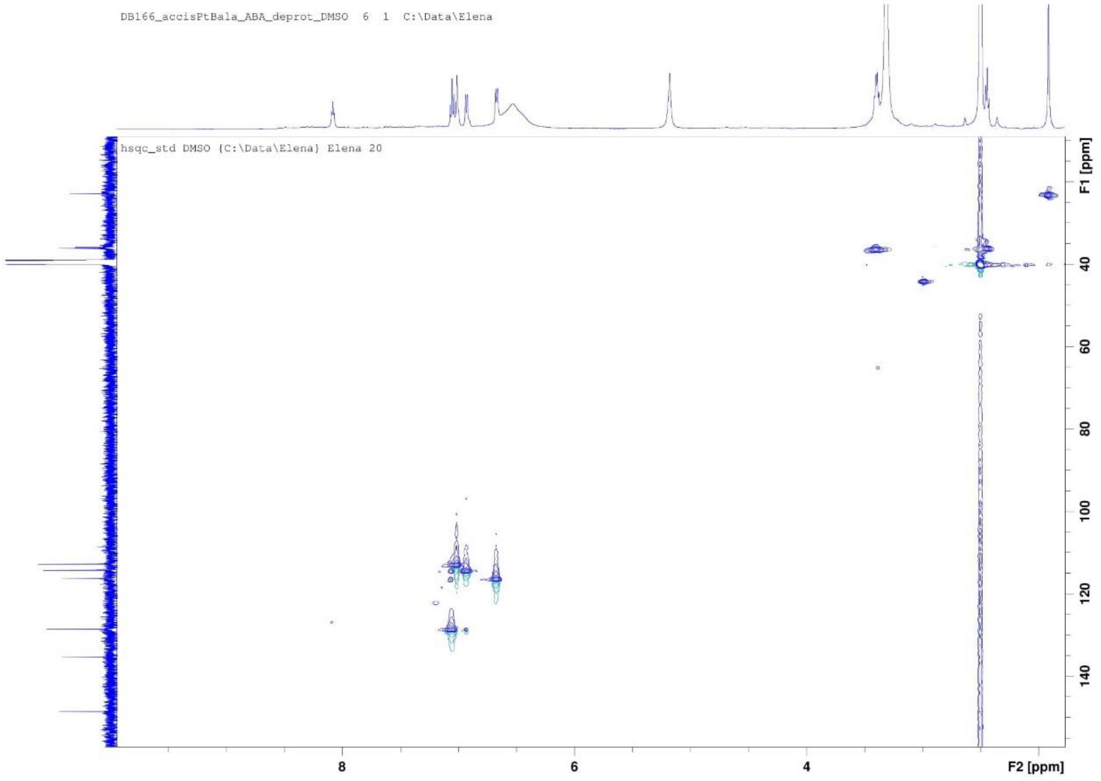


**Figure S31**. [$^{1}$H, $^{13}$C] HSQC NMR spectrum of complex **8**.

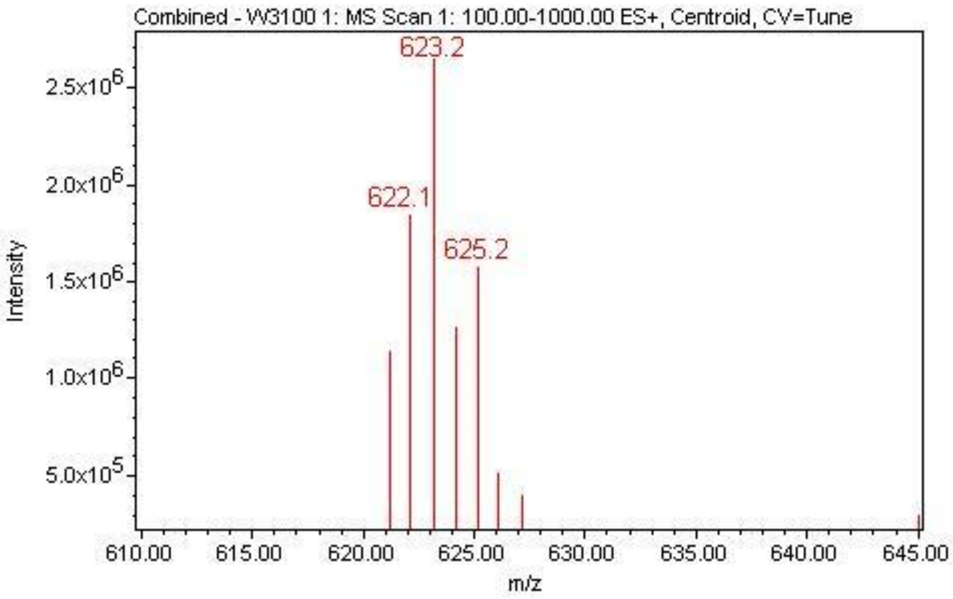


**Figure S32**. ESI-MS spectrum of complex **9**.

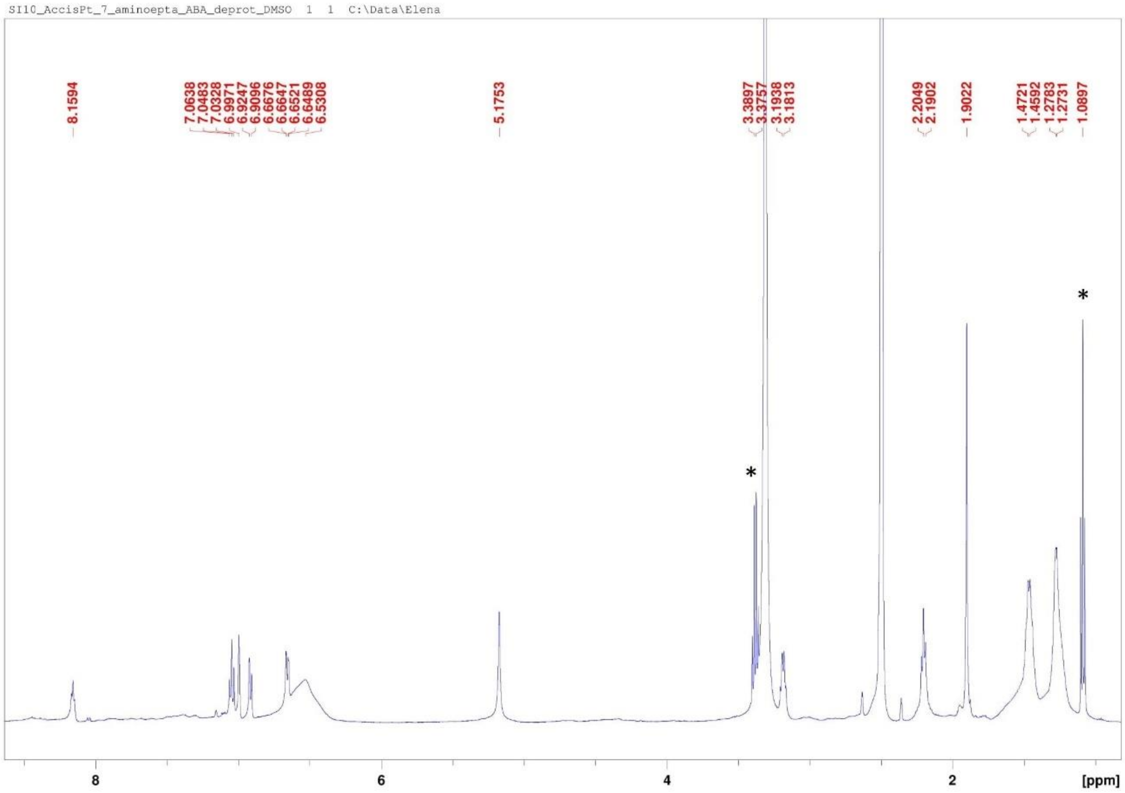


**Figure S33**. $^{1}$H NMR spectrum of complex **9**. The signlas of residual diethyl ether are visible (*).

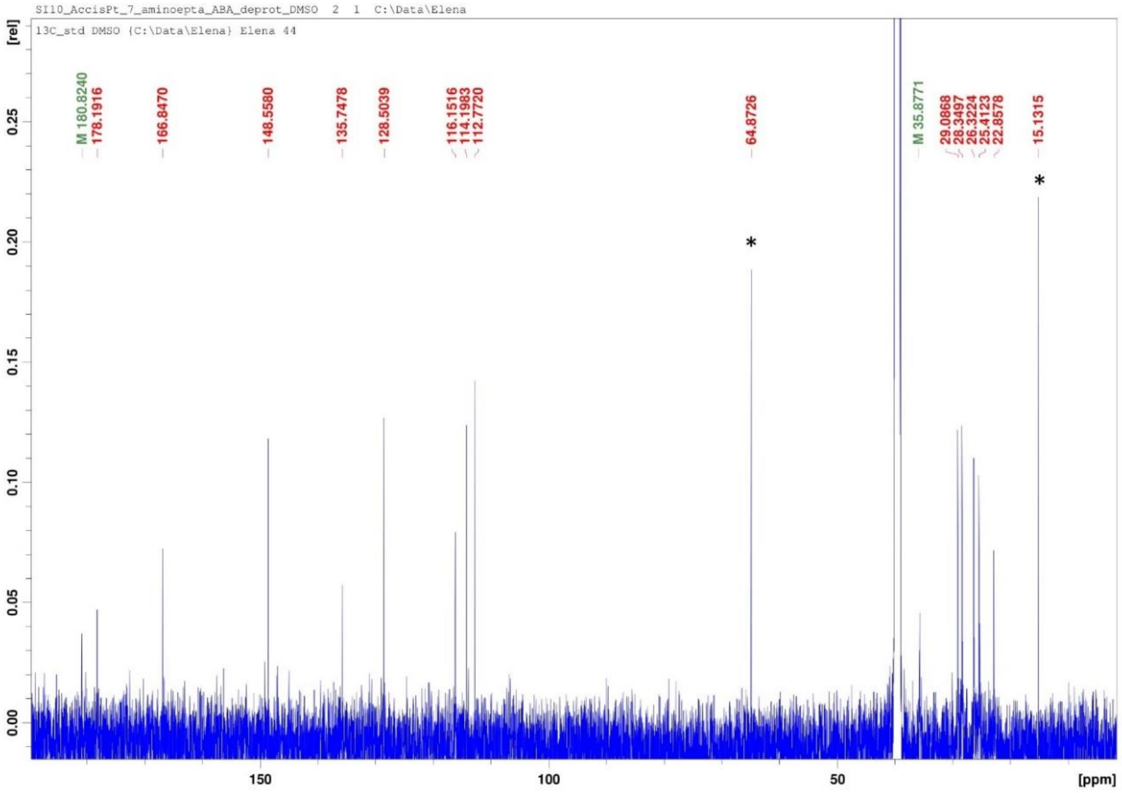


**Figure S34**. $^{13}$C NMR spectrum of complex **9**. The signlas of residual diethyl ether are visible (*).

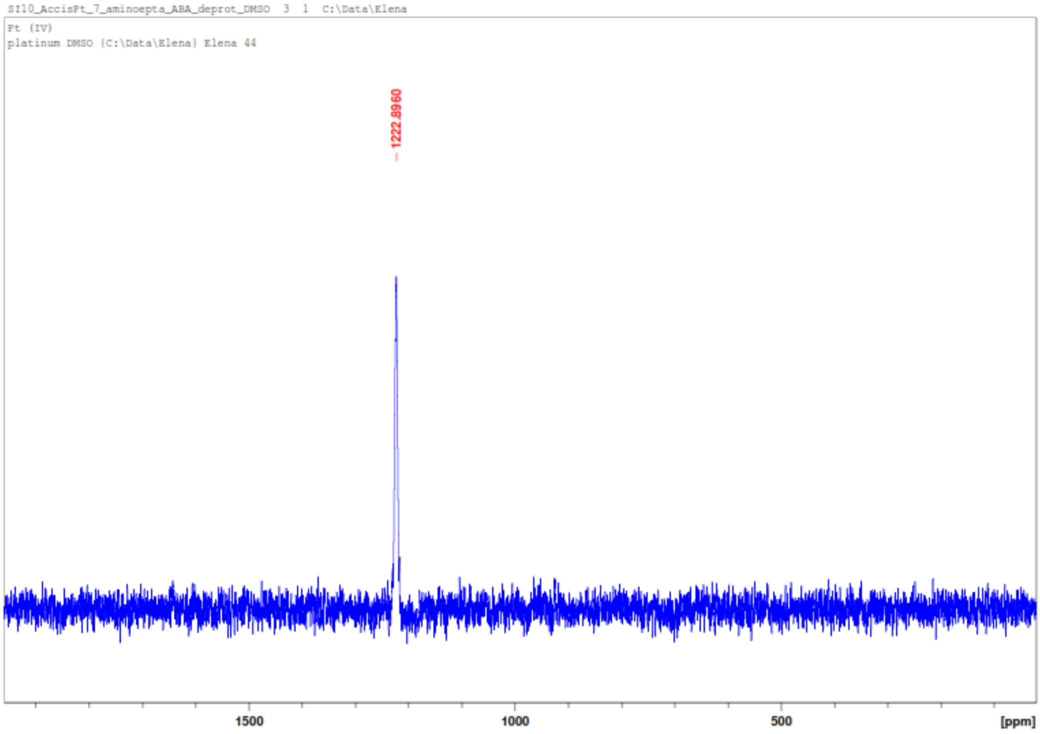


**Figure S35**. $^{195}$Pt NMR spectrum of complex **9**.

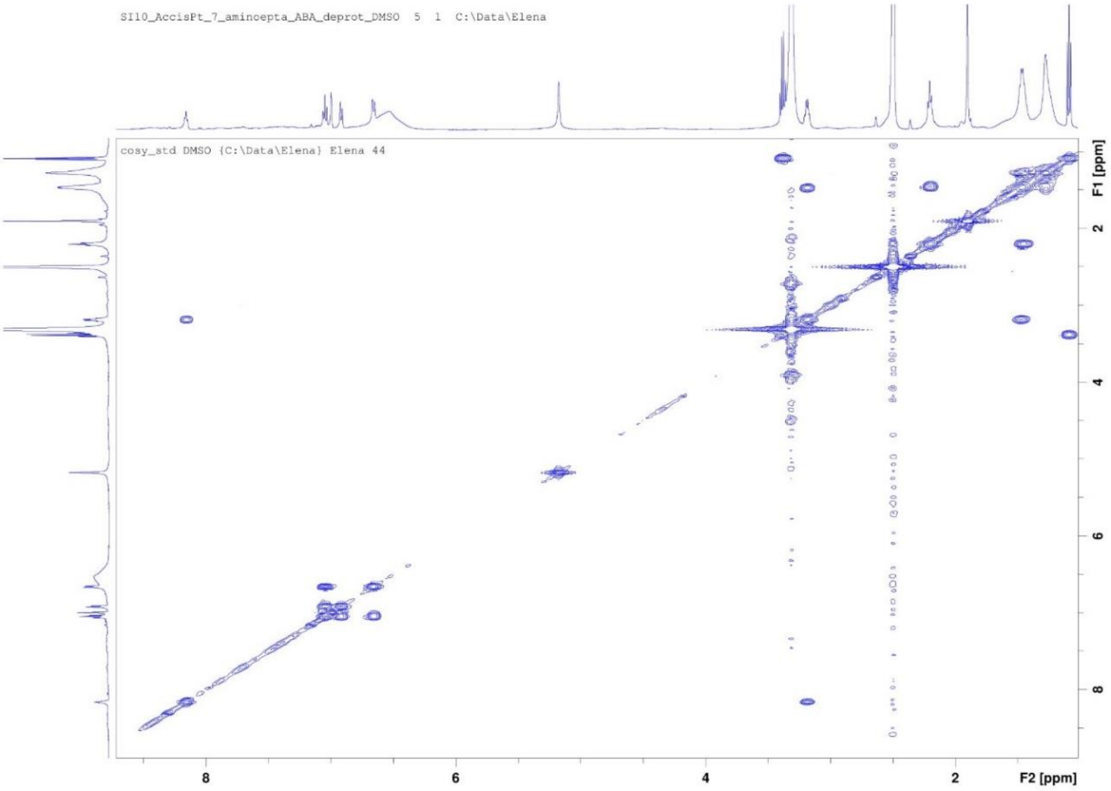


**Figure S36**. [$^{1}$H, $^{1}$H] COSY NMR spectrum of complex **9**.

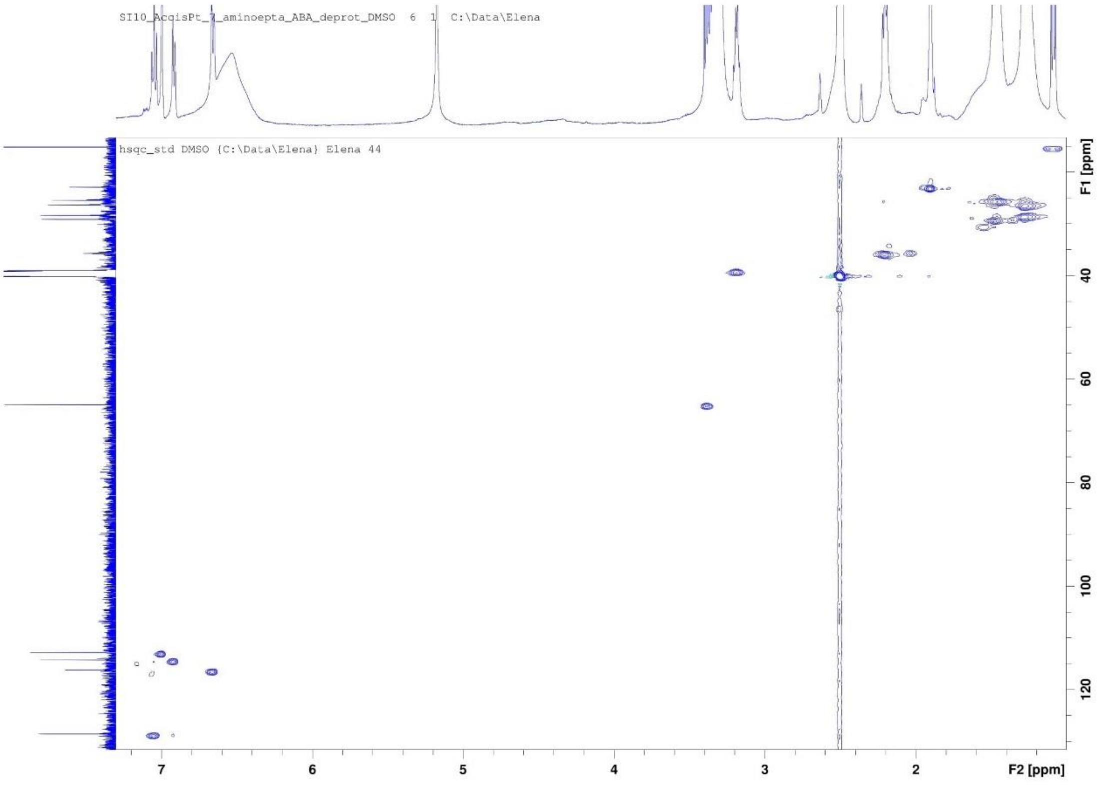


**Figure S37**. [$^{1}$H, $^{13}$C] HSQC NMR spectrum of complex **9**.
